# Highly tail-asymmetric lipids interdigitate and cause bidirectional ordering

**DOI:** 10.1016/j.jlr.2025.100797

**Published:** 2025-04-04

**Authors:** Tugba N. Ozturk, Thomas J. Ferron, Wei He, Benjamin Schwarz, Thomas M. Weiss, Nicholas O. Fischer, Amy Rasley, Timothy S. Carpenter, Catharine M. Bosio, Helgi I. Ingólfsson

**Affiliations:** 1 Biosciences and Biotechnology Division, Physical and Life Sciences Directorate, Lawrence Livermore National Laboratory, Livermore, CA, USA; 2 Material Science Division, Physical and Life Sciences Directorate, Lawrence Livermore National Laboratory, Livermore, CA, USA; 3 Laboratory of Bacteriology, Rocky Mountain Laboratories, Division of Intramural Research, National Institute of Allergy and Infectious Diseases, National Institutes of Health, Hamilton, MT, USA; 4 SLAC National Accelerator Laboratory, Stanford Synchrotron Radiation Lightsource, Menlo Park, CA, USA

**Keywords:** *Francisella tularensis*, lipids, molecular dynamics, phospholipids, phospholipid tail asymmetry, tail asymmetry

## Abstract

Phospholipids form structurally and compositionally diverse membranes. A less studied type of compositional diversity involves phospholipid tail variety. Some phospholipids contain two acyl tails which differ in length. These tail-asymmetric lipids are shown to contribute to temperature sensitivity, oxygen adaptability, and membrane fluidity. Membranes of a highly virulent intracellular bacterium, *Francisella tularensis*, contain highly tail-asymmetric 1-lignoceroyl-2-decanoyl-sn-glycero-3-phosphatidylethanolamine (XJPE) lipids which were previously shown to inhibit inflammatory responses in host cells. XJPE tails have unusually high asymmetry, and how they contribute to membrane properties on a molecular level is unknown. Here, we use small angle X-ray scattering and molecular dynamics simulations to investigate how varying XJPE ratios alters properties of simple membranes. Our results demonstrate that at high concentration they promote liquid-to-gel transition in otherwise liquid membranes, while at low concentration they are tolerated well, minimally altering membrane properties. In liquid membranes, XJPE lipids dynamically adopt two main conformations; with the long tail extended into the opposing leaflet or bent-back residing in its own leaflet. When added to both leaflets XJPE primarily adopts an extended confirmation, while asymmetric addition results in more bent-back orientations. The former increases tail ordering and the latter decreases it. XJPE tails adopt different conformations that induce composition- and leaflet-dependent bidirectional effect on membrane fluidity and this suggests that *Francisella tularensis* could use tail asymmetry to facilitate vesicle fusion and destabilize host cells. The effect of tail-asymmetric lipids on complex membranes should be further investigated to reveal the regulatory roles of high tail asymmetry.

## Graphical abstract

**Figure:**
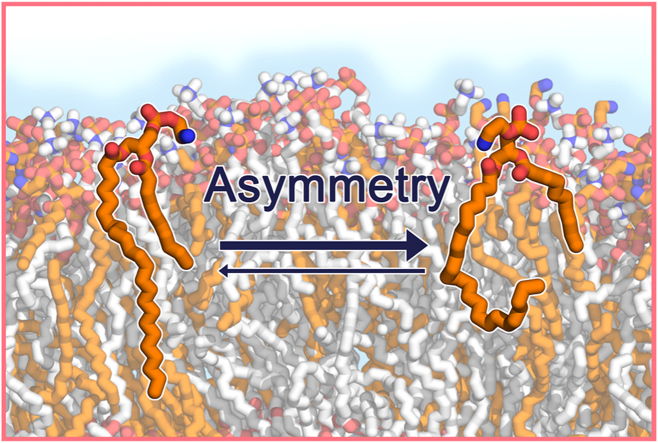

*Francisella tularensis* (*Ft*) is a gram negative, intracellular bacterium and the causative agent of tularemia. It is highly infectious and transmitted by several arthropod vectors, by handling infected material, or inhaling contaminated droplets. Tularemia is treatable with antibiotics, but timely intervention is challenging as symptoms are often delayed until the infection is well established. The *Ft* live vaccine strain is the only available vaccine to protect against tularemia; however, it is inefficient against aerosol infection and is not approved by the Food and Drug Administration (1). The inhalation of *Ft* can cause an atypical pneumonia known as pneumonic tularemia in which the bacteria can infect the lungs and replicate without being detected during the early phases of infection (2). In addition, *Ft* is classified as a category A select agent and potential biological threat by the Centers for Disease Control and Prevention (3). Therefore, there is a need for novel therapeutics and vaccines directed against *Ft*.

In an effort to identify novel virulence factors that may serve as targets for new therapeutics, we identified the lipid fraction of the bacteria as a potent immunomodulator in the host cells, capable of dampening inflammatory responses against a variety of toll-like receptor agonists, as well as responses elicited by viral infection (4, 5, 6). One of the components responsible for this immune modulation was unique phospholipids present in the *Ft* membrane (5). The majority of these lipids consisted of a very long carbon chain, for example, 20–24 carbons, paired with a short carbon chain, for example, 8–12 carbons. Subsequently, we synthesized the most abundant of these tail-asymmetric phospholipids, *1-lignoceroyl-2-decanoyl-sn-glycero-3-phosphatidylethanolamine* (PE 24:0/10:0), referred to herein as XJPE. Independent of other lipid components, XJPE inhibits cytokine secretion in primary human and mouse cells (5), leading us to question how fully saturated, very long–chain lipids with high tail-asymmetry behaves in, and impacts the properties of, eukaryotic membranes as a potential contributing mechanism to the observed immunomodulatory action.

Most phospholipids, including phosphatidylethanolamines, are composed of two acyl tails linked via an ester linkage to the *sn1* and *sn2* positions of a glycerol. The acyl tail at position *sn1* is often saturated (commonly 16:0–18:0) and the one at position *sn2* can be saturated, monounsaturated, or polyunsaturated (commonly 18–22 carbons) (7). These two acyl tails are usually similar in length. Further, in most mammalian membranes the difference in length between two tails of phospholipids is no more than 4–6 carbon atoms, and the *sn2* tail, in such cases, is polyunsaturated (8, 9, 10). Some nonphospholipid membrane components contain tails with length asymmetry but not to the extent seen in *Ft*. For example, sphingolipids often contain one sphingosine tail (18 carbons) and one very long chain (>20 carbons) which can be saturated or contain double bonds. The contribution of these very long chain–containing sphingolipids to membrane structure has been previously investigated but primarily in the context of higher order lipid domains in the background of an otherwise normal fluid bilayer model or specialized biological structures such as extracellular vesicles (11). High asymmetry phospholipids have also been studied, focusing on phosphatidylcholine (PC 18:0/10:0) lipids, as they are an important constituent of the fission yeast *Schizosaccharomyces japonicus* (8, 9, 10) and the baker’s yeast *Saccharomyces cerevisiae* membranes (12). In addition to PC 18:0/10:0 lipids, other lipids with a lesser degree of tail asymmetry such as PC 14:0/18:0 (13, 14) have been studied. In contrast to these well studied lipids, to our knowledge, the molecular mechanisms of membranes composed primarily of lipids with fully saturated and highly asymmetric tails (i.e., 14 carbon atom length difference) have not been pursued nor has the effect of lipids with this degree of asymmetry on normal membranes been investigated.

Here, we combine small-angle X-ray scattering (SAXS) experiments with all-atomistic (AA) and coarse-grained (CG) molecular dynamics (MD) simulations to investigate how a fully saturated, highly tail-asymmetric PE lipid, XJPE (24:0/10:0), behaves and impacts the properties of simple membranes composed of 1,2-dioleoyl-sn-glycero-3-phosphatidylcholine (DOPC) (di18:1), 1,2-dilignoceroyl-sn-glycero-3-phosphatidylcholine (DXPC) (di24:0), or 1-palmitoyl-2-oleoyl-sn-glycero-3-phosphatidylcholine (POPC) (16:0/18:1) lipids. The presented data clearly demonstrate that the addition of tail-asymmetric XJPE lipids creates or enhances interdigitation in simple membranes, meaning that lipid tails penetrate beyond the bilayer midplane (7). We also show that at high concentrations, XJPE lipids induce gel formation in otherwise liquid membranes, while at lower concentrations they modestly alter membrane properties such as thickness, area per lipid, and area compressibility modulus. Finally, we discuss how the impact of tail-asymmetric lipids changes when they are incorporated asymmetrically into a single leaflet of the membranes.

## Materials and Methods

### Liposome preparation

DOPC, DXPC, XJPE, and 1,2-dioleoyl-sn-glycero-3-phosphatidylethanolamine (DOPE) were purchased from Avanti Polar Lipids (Alabaster, AL). For liposome preparation, lipids dissolved in chloroform were mixed in clean glass vials according to the pre-defined molar ratios (0:100, 10:90, 20:80, 40:60, and 80:20 XJPE:DOPC). Chloroform was evaporated under a stream of nitrogen gas during gentle agitation to form a thin lipid film on the wall of the vial. The lipid films were dried further under vacuum for at least 2 h to ensure the complete removal of residual solvent. To form liposomes, the dried lipid films were rehydrated with phosphate buffered saline (137 mM NaCl, 2.7 mM KCl, 10 mM Na_2_HPO_4_, and 1.8 mM KH_2_PO_4_) to a lipid concentration of 5 mg/ml, vortexed for 1 min, and heated to 55°C using a Thermomixer R (Eppendorf, Hamburg, Germany) immediately prior to extrusion. All liposome extrusions were conducted using a syringe extruder fitted with a 200 nm polycarbonate membrane (according to manufacturer’s instruction, Avanti Polar Lipids, Alabaster, AL) while maintaining the syringe heat block at 55°C during the extrusion process. The lipid suspension was passed through the extruder for 11 cycles to form liposomes. After extrusion, liposomal formulations were stored at room temperature (∼25°C) for up to 5 h prior to characterization. The size distribution and polydispersity of the liposomes were measured in triplicates using dynamic light scattering (DLS) on a Zetasizer Nano ZS 90 (Malvern Instruments, Orsay, France) with a 173° scattering angle at 25°C. The liposomes were subsequently stored at 4°C until SAXS measurements (within 24–48 h).

### SAXS experiments

All SAXS samples were prepared at a concentration of 5 mg/ml and up to 48 h ahead of experiments. Further dilution was performed at the beamline to create suspensions with concentrations of 5, 2, and 1 mg/ml, with the assumption that the original suspension did not lose mass during extrusion. Samples were aliquoted into centrifugal tubes with a volume of ∼30 μl and mounted into a 96-well microplate that was maintained at room temperature, ∼298 K, during experiments. SAXS measurements were conducted at Beamline 4-2 at the Stanford Synchrotron Radiation Lightsource (15). A robotic autosampler transferred each sample to a 1.5 mm diameter quartz capillary where it was exposed to X-rays. The X-ray energy was fixed at 11 keV and scattered light was collected on a 2D detector (Pilatus 1M, Dectris) that was positioned ∼1.5 m away from the sample. The scattering geometry was calibrated using an AgBeh standard prior to measurements. Data were averaged over up to 60 sequential camera frames, each with an exposure time of 1 s, and the sample was continuously oscillated during collection to minimize beam damage. A comparison of the data series was conducted prior to averaging to identify if beam damage had occurred, no instances were observed in this series of experiments. Data were reduced from the original 2D detector images to a 1D line out using SAXSPIPE (16). Briefly, 2D images were azimuthally averaged and corrected for X-ray absorption before SAXS measurements from a buffer sample were subtracted from the liposome data. SAXS modeling was performed in IGOR Pro (version 9.01) using a user-modified version of IRENA (version 2.72) (17). Details on the specific model used in this study can be found in the Supplementary Information.

### AA and CG structural models of XJPE and DXPC lipids

The AA topology file for XJPE (PE 24:0/10:0) was generated with CHARMM (version 48b1) (18) using the *par_all36_lipid.prm* parameter file and a manually generated residue topology file. The CG topology file for XJPE, compatible with the Martini 3 (M3) force field, was generated with the Martini lipid topology generator script (version M3.l01) (19) using the argument ‘-alname XJPE -alhead 'E P' -allink 'G G' -altail 'CC cCCCCC'’. For DXPC (PC di24:0), an AA topology file (dxpc.rtf) was manually generated and the argument ‘-alname DXPC -alhead 'C P' -allink 'G G' -altail 'cCCCCC cCCCCC'’ was used to generate the M3 CG topology files. The AA topology files were then converted to GROMACS topology file format (.itp) with ParmED (version 4.2.2) (https://github.com/ParmEd/ParmEd).

### CG MD simulation setups

The CG simulation systems were generated with the INSert membrane (*insane*) Python script (20) on an 8 nm × 8 nm × 13 nm grid, containing 100 lipids per leaflet solvated with a 0.15 M NaCl solution. Bonded and nonbonded interactions between CG particles were computed using M3 force field parameters (21) and newly refined lipidome (19). The resulting membrane systems were equilibrated in five steps starting with 1,500 steps of energy minimization followed by three equilibration steps, with Berendsen thermostat and barostat (22) set to 310 K and 1 bar, as described in more detail in our established protocols (23). The fifth step of equilibration was 2 ns long and carried out with a velocity-rescale thermostat (24) (the time constant for coupling was set to 1 ps) and a semi-isotropic Parrinello-Rahman barostat (25) (the compressibility and time constant for coupling were set to 3 × 10^−4^ bar^−1^ and 12.0 ps, respectively). Using this setup, we built a series of CG MD simulation systems at 298, 310, 315, and 328 K with varying membrane compositions, as listed in Supplemental Table S1. For all conditions, 4 or 10 independent simulation systems were simulated for 10 μs with GROMACS (version 2023.1) (26). To compute the lateral pressure profiles, we extended the simulations for 1 μs with GROMACS LS (version 4.5.5) (27, 28). Details on the analyses of the CG MD simulation trajectories can be found in the Supplementary Information.

### AA MD simulation setups

The configurations of the equilibrated CG MD simulation systems were converted to AA representations using ezAlign (29). CHARMM36 (C36) force field parameters were used to calculate bonded and nonbonded interactions between AA particles in the simulation systems (30). We applied a 1,000 step energy minimization followed by a three step equilibration protocol, similarly to the protocol suggested by CHARMM-GUI Membrane Builder (31): 1) 250 ps with a 1 fs time step in the NVT ensemble; 2) 125 ps with a 1 fs time step in the NPT ensemble; and 3) 1.5 ns with a 2 fs time step in the NPT ensemble. Notably, we did not apply any positional restraints on lipids during these equilibration steps. In all equilibration steps, the temperature was kept at a set value using a Berendsen thermostat (22) with a time constant for coupling of 1 ps, and the pressure was kept at 1 bar using a semi-isotropic Berendsen barostat (22) (the compressibility and time constant for coupling were set to 4.5 × 10^−5^ bar^−1^ and 5.0 ps, respectively). For the production runs, a Nose-Hoover thermostat (32, 33) and a semi-isotropic Parrinello-Rahman barostat (25) were used to keep temperature and pressure constant. The rest of the simulation parameters are identical to the protocol used by CHARMM-GUI Membrane Builder for GROMACS. Finally, for each condition, four independent membrane systems were simulated for 350 ns using GROMACS (version 2023.1) (26), as listed in Supplemental Table S2. Details on the analyses of the AA MD simulation trajectories can be found in the Supplementary Information.

## Results

### Experimental and computational modeling suggest that at room temperature DOPC membranes remain in the liquid state when mixed with up to 40% XJPE lipids

**Fig. 1:**
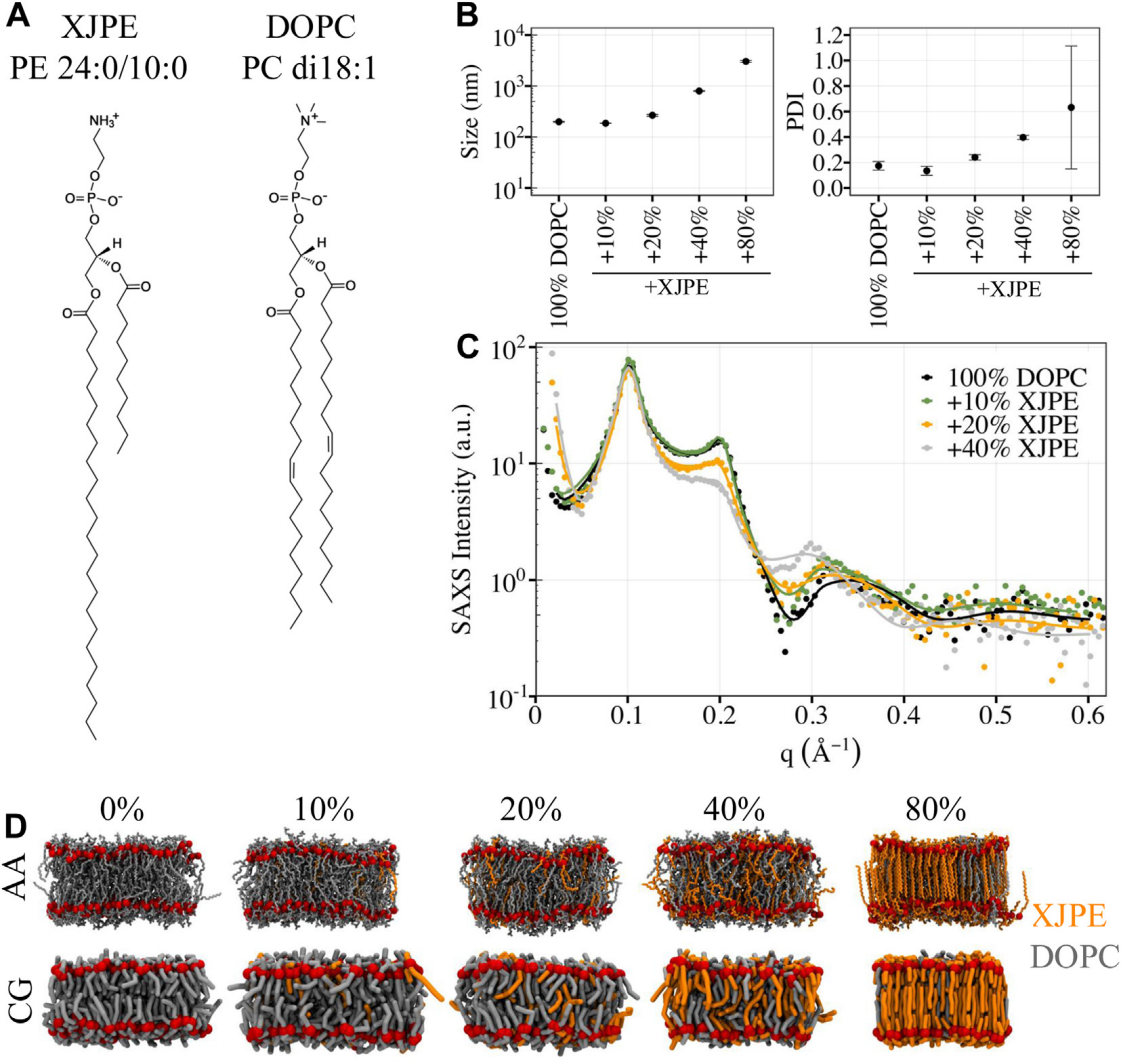
Phase behavior of XJPE-containing DOPC membranes. A: The structures of XJPE and DOPC lipids. B: Average size (left panel) and polydispersity index (PDI; right panel) values for DOPC liposomes containing 0%, 10%, 20%, 40%, and 80% XJPE lipids. The data points are averaged over three independent replicas and the standard deviation around the mean are shown as error bars. C: SAXS intensity profiles of DOPC liposomes containing 0%, 10%, 20%, and 40% XJPE lipids were plotted against the momentum transfer vector (q). Raw and fitted data points are represented as filled circles and solid lines, respectively. D: The snapshots of XJPE:DOPC membranes at the end of 350 ns long AA and 10 μs long CG MD simulations. XJPE and DOPC lipids are colored orange and gray, respectively. Red spheres show the glycerol linker, C21 and C31 atoms in the AA systems and GL1 and GL2 beads in the CG systems. AA, all-atomistic; CG, coarse-grained; MD, molecular dynamics; SAXS, small-angle X-ray scattering; XJPE, 1-lignoceroyl-2-decanoyl-sn-glycero-3-phosphatidylethanolamine.

DOPC (di18:1) lipids, composed of two oleoyl chain tails (both 18 carbon long with a single double bond at the ninth carbon position), are one of the most commonly studied membrane lipids (7) (Fig. 1A). To inspect how fully saturated, tail-asymmetric XJPE lipids impact the properties of DOPC membranes, we prepared XJPE:DOPC liposomes at different compositions using extrusion: 0:100, 10:90, 20:80, 40:60, and 80:20. All liposomes were prepared at 55°C and then equilibrated to room temperature for 3–5 h prior to characterization by DLS (Fig. 1B). As expected, liposomes prepared with 100% DOPC lipids were ∼200 nm in size, with a polydispersity index (PDI) of less than 0.2 (34, 35). Compared to the pure DOPC liposomes, 10:90 XJPE:DOPC liposomes exhibited a similar size and PDI, while those with 20–40% XJPE lipids demonstrated increased size and PDI. At the 80:20 ratio, the amount of pressure required to depress the syringes during extrusion was significantly greater than the other formulations, suggesting that very large vesicles, or vesicle aggregates, were being formed and possibly clogging the filter. Indeed, the resulting particles measured greater than 3,000 nm in diameter with an average PDI above 0.6, indicating that discrete unilamellar vesicles were not uniformly formed.

Next, we conducted SAXS measurements of the prepared liposomes to investigate the structures of XJPE:DOPC membranes. All samples were taken from the same lipid preparation used in DLS and SAXS measurements were conducted at room temperature. Figure 1C shows SAXS data plotted against the momentum transfer vector, q, collected from DOPC liposomes with increasing XJPE concentration. Each sample displayed a similar motif with Bragg peaks near q ≈ 0.1 and 0.2 Å^−1^ convoluted with broad fringes centered around q ≈ 0.15 and q ≈ 0.3 Å^−1^ that can be attributed to the scattering form factor of a liposome bilayer. Lipids have a tendency to assemble into multilamellar vesicles with alternating layers of lipid bilayers and water that gave rise to the Bragg scattering profiles observed in all measured DOPC-based liposomes (36, 37). Interestingly, the scattering minima near q ≈ 0.27 Å^−1^ found in the pure DOPC liposomes (and all DOPE:DOPC liposomes, see Supporting Information) began to disappear as the XJPE concentration increased. This behavior is often attributed to the presence of an asymmetric bilayer membrane and has been observed when the lipid composition of either leaflet is different as well as when the curvature of the inner or outer leaflet causes a variation in headgroup positioning (38, 39). Recently, Frewein *et al*. has further found that tail-asymmetric lipids can induce structural asymmetry through chain interdigitation (14). While it is unclear from the SAXS data alone what causes this phenomenon in our data, given our system, asymmetry in the hydrophobic region could result from the center-of-mass of chain-terminal methyl groups to be offset from the midplane due to interdigitation. A more sophisticated model with a joint analysis of X-ray and neutron scattering may help resolve this question. However, these nuances do not influence the trend in bilayer thickness determined from the SAXS modeling which is the primary metric that we used to validate the simulation data. Full details on the SAXS model are provided in the Supplementary Information. Note that we did not include the data obtained with the 80:20 XJPE:DOPC liposomes in our analysis. The complications during extrusion and the results of the DLS provided enough doubt as to whether the sample consisted of proper vesicles. SAXS data collected from the same solution as used in the DLS further suggested the presence of a more exotic gel-phase compared to our other samples. An example profile can be found in the Supporting Information, but further analysis would be beyond the scope of the present study.

To investigate the structures of XJPE:DOPC membranes at a molecular level, we carried out AA and CG MD simulations at various ratios: 0:100, 10:90, 20:80, 40:60, and 80:20 (Fig. 1D). AA and CG modeling demonstrated consistent liquid-to-gel transitions as the proportion of XJPE lipids increased in DOPC membranes. 80:20 XJPE:DOPC membranes were found to form gels, while those containing 40% or less XJPE remained liquid over the course of simulations conducted at 298 K. It is important to note that the XJPE:DOPC membrane configurations given in Figure 1D show interdigitation between leaflets due to the longer acyl chain of XJPE lipids extending beyond the bilayer center into the opposing leaflet.

### Addition of XJPE lipids into DOPC membranes minimally alters bilayer properties

**Fig. 2:**
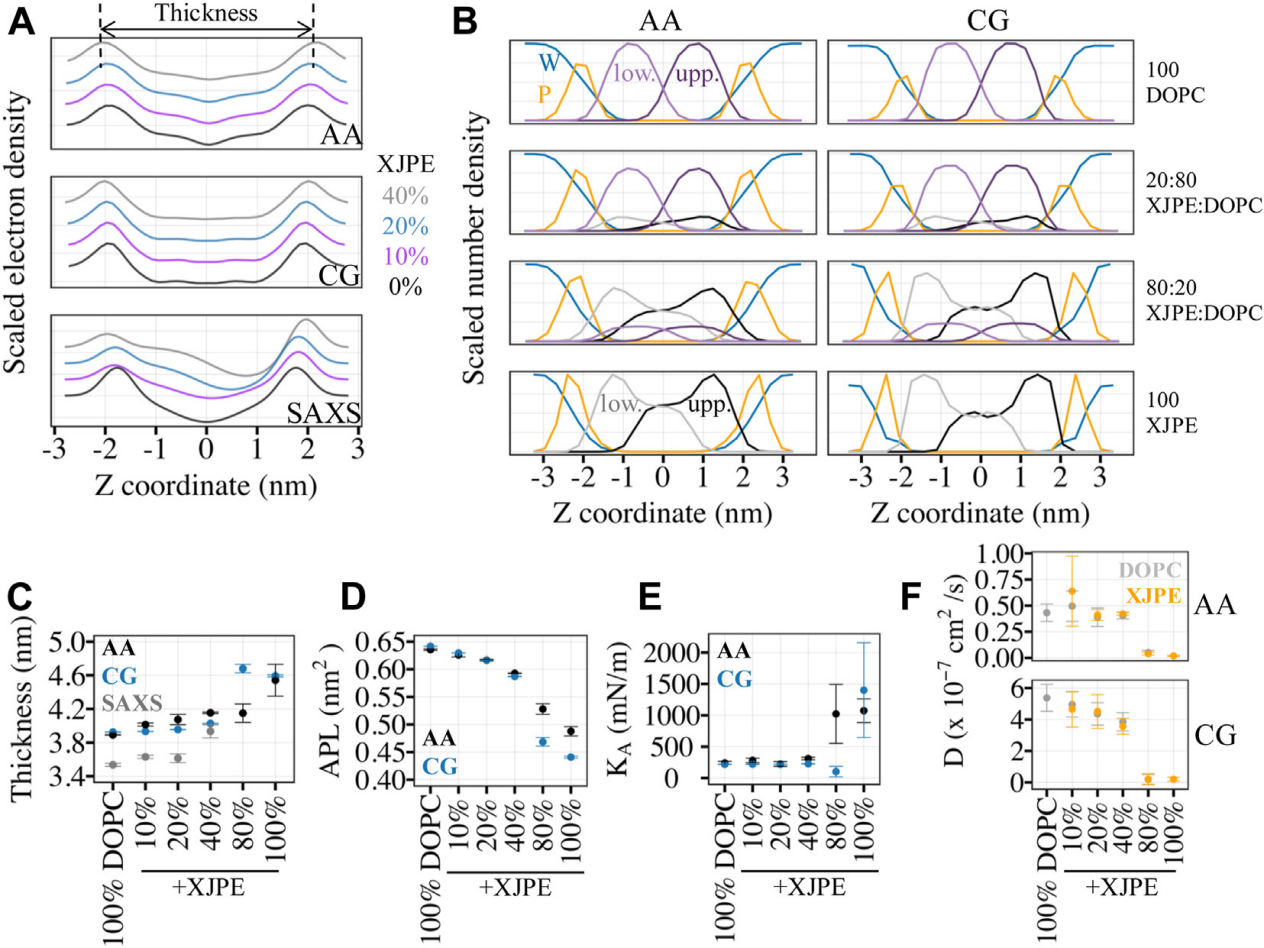
Properties of XJPE:DOPC membranes. A: Shown are the total electron density profiles of XJPE:DOPC membranes along the membrane normal. The top, middle, and bottom panels are profiles generated using AA, CG MD simulations and SAXS measurements, respectively. The colored-lines represent the data for different membrane compositions: black, 0:100 XJPE:DOPC; purple, 10:90 XJPE:DOPC; blue, 20:80 XJPE:DOPC; and gray, 40:60 XJPE:DOPC. B: The number density profiles of water (blue), phosphates (orange), XJPE tails (black, upper leaflet; gray, lower leaflet) and DOPC tails (purple, upper leaflet; light purple, lower leaflet) were computed from the AA (left) and CG (right) MD simulations. C: Membrane thickness, (D) area per lipid (APL), (E) area compressibility modulus (K_A_), and (F) lateral diffusion coefficients (D) were averaged over multiple simulation trajectories for each XJPE:DOPC membrane and plotted against the membrane composition. The standard deviation around the mean was calculated using four independent AA or CG MD simulations for each membrane composition. The membrane thickness was estimated as the distance between two peaks as shown in panel A. Note that the lateral diffusion coefficients are reported as is, without any correction accounting for the faster diffusion observed at the CG level (49). AA, all-atomistic; CG, coarse-grained; MD, molecular dynamics; SAXS, small-angle X-ray scattering; XJPE, 1-lignoceroyl-2-decanoyl-sn-glycero-3-phosphatidylethanolamine.

The total electron density profiles can be modeled using the SAXS data and estimated from the AA and CG MD trajectories. Figure 2A shows the total electron density profiles of XJPE:DOPC membranes, computed using these three methods. The important components of the MD profiles are individually shown in Figure 2B as scaled number densities. A notable feature is the overlap between the densities of lipid tails in opposing leaflets: membranes became more interdigitated as their XJPE proportion increased. Since the XJPE *sn2* tails are too short to reach the membrane midplane the much longer XJPE *sn1* tails from the opposite leaflet have room to penetrate beyond the midplane without causing significant packing defects. The interdigitation resulted in a lower acyl tail density in the membrane interior than pure DOPC membranes. Furthermore, we consistently observed modest changes in all membranes with low XJPE concentrations: a small increase in thickness, a decrease in the area per lipid (APL), and negligible variations in area compressibility moduli (K_A_) and lateral diffusion coefficients (D) (Fig. 2C–F and Supplemental Fig. S1B, C). At high XJPE concentrations, these properties changed quite significantly as the membranes went into gel phases.

We calculated the membrane thickness as the distance between two peaks observed in the electron density profiles. Figure 2C shows that the small increase in membrane thickness was a consistent trend observed with all three methods. For example, for the membranes with 20% XJPE, the observed increases were less than 0.18 nm in all three methods, compared to the thickness of pure DOPC membranes. On average, the experimental estimations were 0.4 (0.3) nm smaller than the MD estimations. The AA and CG trends was the same except for one notable deviation for 80:20 XJPE:DOPC membranes where the liquid-to-gel transition occurred. For high XJPE proportions, we only have computational data as liposomes with 80% XJPE were of poor quality.

The changes in the APL, K_A_, and D were also modest except for the membranes with high XJPE compositions. For low ratios of XJPE, both AA and CG modeling showed slightly decreasing APL with increasing XJPE proportions in the DOPC membranes (Fig. 2D). For example, the APL of 20:80 XJPE:DOPC membranes was about 0.02 nm^2^ lower than the APL of 100% DOPC at both AA and CG resolutions, indicating a small increase in packing due to the incorporation of XJPE. The K_A_ did not change with increasing XJPE proportion up to >40% XJPE in both AA and CG MD simulations (Fig. 2E). For 80:20 XJPE:DOPC membranes, a pronounced increase in K_A_ was observed due to gel formation in the AA MD simulations. In the CG MD simulations, 80:20 XJPE:DOPC membranes still maintained K_A_ comparable to membranes with lower XJPE ratios, possibly due to the presence of small areas/defects with relatively disordered lipids. CG MD simulations resulted in a 15–20% decrease in D of lipids with increasing XJPE ratios in the membranes, while AA MD simulations showed comparable results independent of the lipid species. In both AA and CG models, lateral movement of lipids slowed down significantly for 80:20 XJPE:DOPC and 100% XJPE membranes, as expected since these membranes were in or very close to gel state. Finally, in the simulations, XJPE lipids mixed well with DOPC lipids without any aggregation except for the 80:20 XJPE:DOPC gel membranes at 298 K (Supplemental Fig. S4A).

Altogether, the impact of XJPE on the properties of DOPC membranes was found to be mild. For comparison, we checked the same amounts of added DOPE lipids which have the same symmetric monounsaturated acyl tails as DOPC and the same headgroup as XJPE. SAXS measurements, presented on Supplemental Fig. S2C, showed that DOPE:DOPC liposomes exhibited an increase in thickness with increasing proportions of DOPEs, similarly to what we observed in XJPE:DOPC liposomes. However, we did not observe any sign of asymmetric membranes in the SAXS profiles, further supporting that what we observed in XJPE-containing liposomes could be due to interdigitation. MD simulations of DOPE:DOPC membranes at the same ratios (Supplemental Figs. S2 and S3) demonstrated that the membrane properties changed comparably. Similar increase in membrane thickness and decrease APL was observed for XJPE:DOPC and DOPE:DOPC (Fig. 2 and Supplemental Fig. S3). However, the changes in phase transitions and interdigitation were only observed in the presence of the tail-asymmetric XJPE lipids.

### Addition of XJPE lipids increases interdigitation with only a modest change in DOPC membrane ordering

**Fig. 3:**
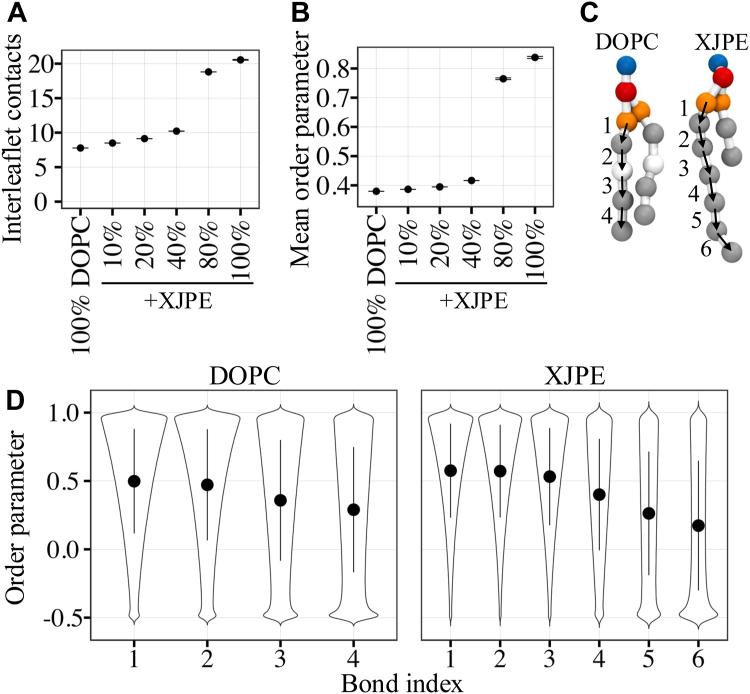
Interleaflet contacts and lipid ordering in XJPE:DOPC membranes. A: Average interleaflet contacts for XJPE:DOPC membranes at different ratios. Error bars show the standard deviation around the mean computed over the course of 4 independent CG MD simulations of each membrane composition. B: Mean order parameter is calculated as an average over all tail bonds of lipids through independent CG MD simulations. C: Tail bond indices are shown for the *sn1* tails of DOPC (left) and XJPE (right) lipids. D: The distributions of the order parameter of each bond shown in panel C are given for DOPC (left) and XJPE (right) lipids using the CG simulations of 20:80 XJPE:DOPC membranes at 298 K. The solid circle and lines represent mean and mean ± standard deviation, respectively. CG, coarse-grained; MD, molecular dynamics; XJPE, 1-lignoceroyl-2-decanoyl-sn-glycero-3-phosphatidylethanolamine.

Qualitatively, both SAXS data and lipids configurations observed through the MD simulations suggest that the leaflets became more interdigitated with increasing XJPE ratios in the membranes. One way to quantify interdigitation is to inspect the overlap between the density profiles of lipid tails from individual leaflets, as shown in Figure 2B. Another way is to calculate the number of interleaflet contacts (contacts between lipid tails from opposing leaflets). Figure 3A shows that more interleaflet contacts were formed as the XJPE ratio increased in the membranes. For example, 40:60 XJPE:DOPC membranes maintained 30% more contacts on average than 100% DOPC membranes. With higher XJPE proportions, gel membranes formed almost twice more interleaflet contacts than the 40:60 XJPE:DOPC membranes in the liquid state. We also observed that tail ordering increased modestly when the XJPE ratio increased up to 40%, while at high XJPE ratios, in gel phase a sharp increase in tail ordering was present (Fig. 3B).

The XJPE *sn1* tail is a fully saturated 24 carbon-long acyl tail which is represented with 6 tail beads in the CG model (Fig. 3C right panel). Similarly, the DOPC *sn1* tail contains 18 carbon-long monounsaturated acyl tail represented with 4 CG beads (Fig. 3C left panel). Each bond connecting these tail beads are numbered and marked with black arrows in Fig. 3. We calculated the order parameter distribution for each XJPE bond using 4 independent 10 μs long CD MD simulations of 20:80 XJPE:DOPC membranes and compared them with the distributions obtained for the DOPC *sn1* tail bonds (Fig. 3D). These distributions revealed that the first four bonds of XJPE *sn1* tails were on average more ordered than DOPC tails. However, the last two XJPE beads were relatively more disordered and the disordered tail configurations were more frequently visited. As observed from the set of control simulations of DOPE:DOPC membranes, neglectable effects was seen on interleaflet contacts and only a similar modest increase in order parameters was observed in the presence of DOPE lipids (Supplemental Fig. S5), suggesting that changes were solely caused by tail asymmetry.

### Addition of XJPE lipids increases interdigitation and decreases lipid ordering in DXPC membranes

In an earlier work, we showed that *Ft* XJPE can drive the suppression of inflammatory responses in both human and mouse cells (5). These experiments were conducted with *Ft* membrane extracts and were also verified with synthetic 80:20 XJPE:DXPC liposomes, where DXPC has two long saturated tails (PC di24:0). Here, we examined the molecular structures of XJPE:DXPC membranes using CG MD simulations at 100:0, 0:100, 90:10, and 80:20 ratios (Supplemental Fig. S9). We expected XJPE:DXPC membranes to be in the gel state since the melting temperature of DXPC is reported as 353 K (40). Therefore, we carried out the simulations at three different temperatures: 310, 315, and 328 K. Indeed, 0:100, 90:10, and 80:20 XJPE:DXPC mixtures formed gel membranes (Supplemental Fig. S9A). These membranes had low APL (<0.53 nm^2^), very slow lipids (<3 × 10^−10^ cm^2^/s), high K_A_ (>980 mN/m), and high tail order (Supplemental Fig. S9E–H). 80:20 XJPE:DXPC membranes occasionally had a few XJPE lipids with disordered tail configurations, yet the mean tail order was still larger than 0.65 between 310 and 328 K. One hundred percent XJPE membranes were also in the gel state at 310 and 315 K, with membrane properties comparable to the membranes with other ratios of XJPE (Supplemental Fig. S9). However, at 328 K, pure XJPE membranes were melted; we observed significant increases in APL and in lateral diffusion coefficients of lipids, along with noticeable decreases in K_A_ and tail order.

The addition of XJPE caused significant decreases in the thickness of DXPC membranes, independent of the temperature (Supplemental Fig. S9D). For example, 100% DXPC membranes had a thickness of 6.5 nm at 310 K and the addition of 80% XJPE at this temperature resulted in 4.6 nm thick membranes. The same decrease was observed at 315 and 328 K. Moreover, XJPE:DXPC membranes were highly interdigitated; they maintained three times the number of interleaflet contacts than 100% DXPC membranes (Supplemental Fig. S9C, I). Finally, we carried out a series of POPE:DXPC membrane simulations at the same three temperatures as controls (Supplemental Fig. S9). POPE:DXPC were all in the liquid state, with properties comparable to 100% POPE membranes. Compared to XJPE:DXPC membranes, they were thinner, less interdigitated (maintaining four times fewer interleaflet contacts) and more disordered at all temperatures. This further demonstrates that tail asymmetry introduces interdigitation.

### The effect of asymmetric incorporation of XJPE lipids on the properties of simple membranes

**Fig. 4:**
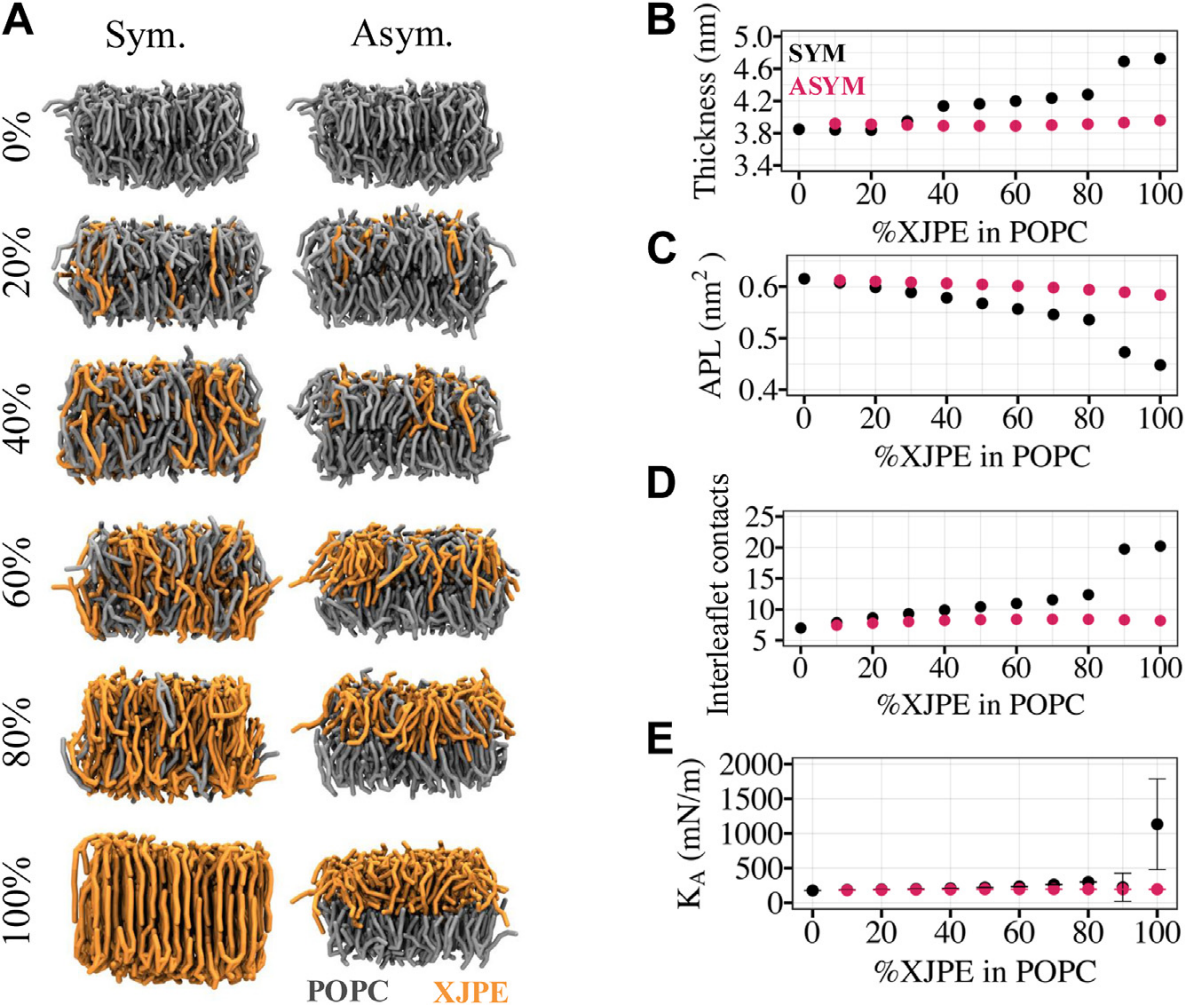
Properties of XJPE:POPC membranes. A: The XJPE:POPC membranes at different compositions are shown where XJPE is added either symmetrically into both leaflets (left column) or asymmetrically only into one leaflet (right column). This is a subset of all systems investigated, Supplemental Fig. S6 includes representative snapshots of all XJPE:POPC membranes. The mean values of (B) membrane thickness, (C) area per lipid, (D) interleaflet contacts, and (E) area compressibility modulus are computed over 10 independent CG MD simulations and plotted against the percentage of XJPE lipids in the membranes. The black and pink data points represent the symmetric and asymmetric membrane mixtures, respectively. In panel B-D, the error bars are not shown as they are smaller than 0.1 nm, 0.002 nm^2^ and 0.25 with the given order. In panel E, the shown error bars represent the standard deviation around the mean. CG, coarse-grained; MD, molecular dynamics; XJPE, 1-lignoceroyl-2-decanoyl-sn-glycero-3-phosphatidylethanolamine.

So far, we presented data on DOPC and DXPC membranes with XJPE lipids incorporated symmetrically, that is, into both leaflets of the membranes. In these symmetric XJPE-containing membranes, we showed that the addition of XJPE mildly changed properties such as thickness and APL. In this section, we explored how membrane properties changed when XJPE lipids were added asymmetrically (to a single leaflet of the membrane) in simple membranes composed of DOPC or POPC lipids. The impact of asymmetrically added lipids with high tail asymmetry is particularly important since it could be how *Ft* vesicles fuse into host membranes. For this purpose, we carried out a series of CG MD simulations of symmetric and asymmetric membranes containing varying ratios of XJPE lipids (Supplemental Table S1). Figure 4A includes the resulting configurations of a subset of XJPE:POPC membranes (see Supplemental Fig. S6 for all membrane conditions) and Supplemental Fig. S7A for XJPE:DOPC membrane conditions. All CG MD simulations presented in this section were carried out at 310 K.

Symmetric XJPE:POPC membranes were found to form gels at high XJPE proportions, apparent from the significant increase in membrane thickness and decrease in APL (Fig. 4B,C). At 30% or less XJPE ratios, the observed changes in K_A_ were minimal, interleaflet contacts and thickness showed mild increases, and APL decreased with respect to those found in the pure POPC membranes (Fig. 4B–E). In asymmetric XJPE:POPC membranes (added in the same molar ratio), these changes were less pronounced. The asymmetric addition of XJPE did not cause gel formation even at high proportions, unlike for the symmetric incorporation of XJPE. The symmetric and asymmetric addition of XJPE resulted in the similar changes in DOPC membranes (Supplemental Fig. S7). Overall, our data demonstrated that XJPE lipids can be incorporated both symmetrically and asymmetrically with only modest changes to these measured bulk bilayer properties.

### XJPE tails adopt different conformations in symmetric and asymmetric membranes

**Fig. 5:**
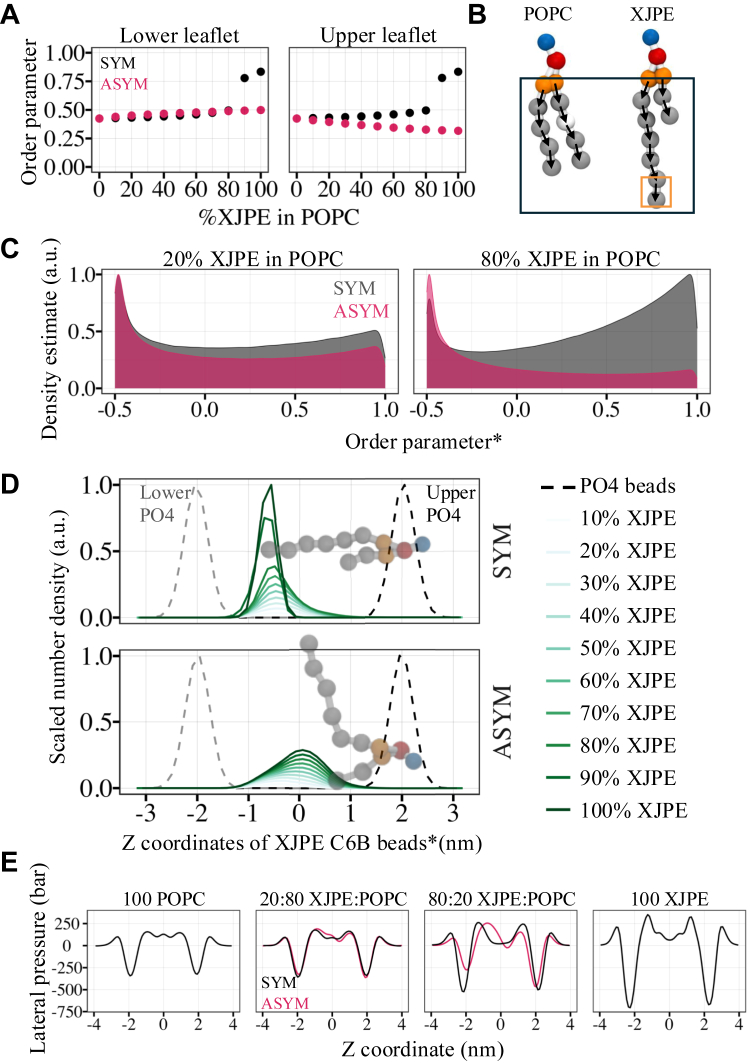
Lipid ordering in XJPE:POPC membranes. A: Mean order parameter is plotted against the membrane composition. The data for symmetric and asymmetric XJPE:POPC membranes are shown in black and pink, respectively. The mean order parameter averaged for all the lipids was computed for those in the lower (left) and upper (right) leaflets separately. In the asymmetric case XJPE is added to the upper leaflet only. B: The ball and stick representation of CG POPC (left) and XJPE (right) lipids. The black frame shows the tail bonds used to calculate the mean order parameters presented in panel A, while the orange frame highlights the last tail bond in the XJPE’s *sn1* tail that is used to calculate the order parameter distribution shown in panel C. C: The scaled density of order parameter was calculated for the last bond of XJPE’s *sn1* tail using the CG MD simulations of 20:80 and 80:20 XJPE:POPC at 310 K. The data for symmetric (black) and asymmetric (pink) XJPE:POPC membranes are shown with 20% transparency. D: The scaled number density of the last tail bead was plotted for the *sn1* tail of XJPE lipids in the upper leaflets of symmetric (top) and asymmetric (bottom) XJPE:POPC membranes. A representative XJPE lipid configuration is scaled properly for the calculated density profiles and is shown transparently on the graphs. The solid lines represent the number density of tail beads and are colored from light to dark green for 10:90, 20:80, 30:70, 40:60, 50:50, 60:40, 70:30, 80:20, 90:10, and 100:0 XJPE:POPC as labeled, whereas the dashed lines represent the phosphate beads in the lower (gray) and upper (black) leaflets from the CG MD simulations of 20:80 XJPE:POPC membranes. In panels C and D, the star symbols in the *x*-axis label highlight that the shown data is computed only for the XJPE lipids in the upper leaflet of the membranes. E: Lateral pressure profiles for 0:100, 20:80, 80:20, and 100:0 XJPE:POPC membranes, given from left to right, are plotted against the membrane normal and are colored black and pink for the symmetric and asymmetric membranes, respectively. CG, coarse-grained; MD, molecular dynamics; XJPE, 1-lignoceroyl-2-decanoyl-sn-glycero-3-phosphatidylethanolamine.

The symmetric addition of XJPE lipids in DOPC or POPC membranes mildly increased tail ordering (Figs. 3, 5A, Supplemental Fig. S5). To inspect how tail ordering changes when XJPE lipids were incorporated asymmetrically into POPC membranes, we computed the order parameter averaged over all tail beads of both lipid species and plotted. Figure 5A shows that the lipid tails became more ordered, with the average order parameter increasing from 0.43 (0:100 POPC) to 0.50 (80:20 XJPE:POPC), with increasing XJPE ratios in symmetric membranes. When XJPE lipids were added to the upper leaflet only (ASYM case) the trend was reversed for the lipid in the upper leaflet, and the lipid tails became less ordered as the XJPE proportion increases (Fig. 5B). For example, the mean order parameter was 0.33 in the asymmetric 80:20 XJPE:POPC membranes as opposed to 0.40 in the asymmetric 20:80 XJPE:POPC membranes. However, the lipids in the lower leaflet showed the same increase as with the symmetric XJPE addition.

To explore the tail ordering in more detail, we analyzed the distribution of instantaneous order parameters computed only for the last bond of XJPE *sn1* tails in the upper leaflet of membranes at 20:80 and 80:20 XJPE:POPC ratios (Fig. 5C). This analysis showed that in both symmetric and asymmetric membranes, XJPE *sn1* tails dynamically adopted conformations where they can be aligned or anticorrelated with the membrane normal. Aligned with the membrane normal, tails exhibited higher order parameter, and they extended toward the opposing leaflet, increasing interleaflet contacts and therefore interdigitation. In the anticorrelated conformations, tails were in a bent-back orientation where the end of the tail faced toward its own leaflet and became, on average, more disordered (Fig. 5A). In the asymmetric membranes, XJPE *sn1* tails were more disordered than in the symmetric membranes, adopting the bent-back conformation more frequently with increasing XJPE proportion.

To quantify how systematically XJPE tails adopted the bent-back conformation, we examined the distribution of last tail bead positions along the membrane normal for all symmetric and asymmetric membranes (Fig. 5D). The XJPE *sn1* tails were analyzed only for the upper leaflet lipids so that if the tail was in the extended conformation, it penetrated beyond the bilayer midplane (z = 0 nm) and therefore its last bead was expected to be closer to the phosphates in the lower leaflet (gray dashed lines). If the tail adopted a bent-back conformation, the last bead was expected to be closer to the phosphates in the upper leaflet (black dashed lines), that is, pointing toward its own leaflet.

The top panel in Figure 5D shows that for the symmetric XJPE membranes the *sn1* tail ends had significantly narrower distributions with 90% or more XJPE (where the membrane is in gel phase). In symmetric liquid membranes, XJPE lipids sometimes adopted the bent-back conformations, although they mostly had their *sn1* tails in the extended orientation. In the asymmetric membranes, the distributions were more spread out and their average position shifted toward the upper leaflet as the XJPE proportion increased in the membranes, with more bent-back orientation at high XJPE concentrations. Our data clearly demonstrate that while XJPE lipids in simple membranes were dynamic, adopting both extended and bent-back conformations in all membrane conditions, one conformation can be favored depending on the ratio symmetry of XJPE incorporation. The change in tail conformations in the asymmetric membranes deserves particular attention as the fusion of bacterial membrane vesicles is likely to disrupt the host membrane starting from a single leaflet. Note that the bent-back conformation was also observed in AA MD simulations of interdigitated membrane mixtures containing lipids with less pronounced tail asymmetry compared to *Ft* XJPE (14).

Finally, we computed the lateral pressure profiles for pure POPC and XJPE membranes as well as the asymmetric and symmetric 20:80 and 80:20 XJPE:POPC membranes. The lateral pressure profile show the three typical features: a repulsive component due to lateral hydration pressure on the headgroups; an attractive component located at the water-lipid interface; and a repulsive component corresponding to steric interactions of the acyl tails (41, 42, 43). We observed the most pronounced features in the profile of pure XJPE membranes, which were in the gel phase (Fig. 5E). The profiles of both symmetric and asymmetric 20:80 XJPE:POPC membranes were comparable to the POPC membrane’s profile. The difference between the two profiles was in the region corresponding to the repulsive component located at the acyl tails. This is expected due to the different dominant conformations that XJPE *sn1* tails adopted in these membranes. The difference in the pressure profiles were more pronounced with an increasing percentage of XJPE lipids in the symmetric and asymmetric membranes. Particularly, in the profile of the asymmetric membrane with 80% XJPE, a large change in the membrane-water interface and hydrocarbon regions was observed (i.e., the shifted peak positions and the decreasing peak heights), suggesting a noticeable change in membrane elasticity.

Note that all of these observations also hold for XJPE:DOPC membranes (Supplemental Fig. S8). These results clearly demonstrate that the impact of XJPE lipids on simple membrane properties is overall mild, and this impact can be different depending on the symmetric or asymmetric incorporation of XJPE lipids. The latter is particularly interesting because it can help us understand the effect of *Ft* XJPE lipids on the host membrane at a molecular level. The observed immunomodulatory effect of XJPE lipids raises the question of how *Ft* delivers XJPE lipids and possibly other virulence factors into host cells (5). The long-distance delivery, entry, and trafficking of XJPE into the host membrane could be done by bacterial membrane vesicles. XJPE lipids can modulate changes in membrane properties when they are incorporated into a single leaflet of the host membrane, as required by the incorporation of a bacterial membrane vesicle or via contact-driven phenomena.

## Discussion

In this study, we investigated the impact of the tail asymmetric *Ft* lipid XJPE on simple model membranes. The presented AA and CG MD simulation data, vesicle stability, and SAXS measurements agree well with each other, demonstrating that XJPE can be successfully modelled at different spatiotemporal resolutions. Tail-asymmetric lipids promote interdigitation in DOPC, POPC, and DXPC membranes. XJPE can increase or decrease membrane order depending on the membrane composition and their asymmetric or symmetric presence in the membranes. When added into both leaflets of a membrane at proportions lower than 80%, XJPE lipids induce changes in average membrane properties that are modest and comparable with the changes observed in the presence of DOPE. At high proportions, the symmetric incorporation of XJPE cause gel formation and make vesicle formation difficult, as suggested by the emergence of complex, nonlamellar lipid phases in the SAXS data and by the emergence of liposomes with high PDI. The asymmetric addition of XJPE lipids, even at high proportions, does not cause liquid-to-gel transition. Regardless of their incorporation, XJPE adopts two notably different conformations: one where the fully saturated very long *sn1* tail bends back toward its own leaflet and the other where the long tail extends into the opposing leaflet, increasing ordering of the entire membrane. The presence of the much shorter *sn2* tail supports the interdigitation of longer *sn1* tails from XJPE lipids in the opposing leaflet minimizing disruption of the lipid packing. The dominant conformation is determined by the amount of XJPE in the membrane, whether they are added asymmetrically into one leaflet or symmetrically into both leaflets (Fig. 5D), and possibly the lipid composition of the host membrane. Depending on the dominant conformation of *sn1* tails, XJPE can show bidirectional effect on tail ordering; in the extended conformations it increases membrane order and in the bent-back conformations, it increases disorder in tails. Membrane properties including lateral stress profiles are readily impacted with the asymmetric incorporation of XJPE lipids in DOPC or POPC membranes.

*Ft* infection is not an isolated example of a very long–chain saturated fatty acid presence in mammalian membranes. These fatty acids can be synthesized in the endoplasmic reticulum by a specific family of enzymes (44) or obtained from diet (45) and play key roles in cell signaling and membrane regulation. Recent reviews suggest that very long–chain saturated fatty acids impact eye and brain function (46) as well as better aging (47). Recent data on tail-asymmetric lipids show that they alter the bending dynamics of membranes (13) and mediate temperature sensitivity (10), oxygen adaptability (9), and membrane fluidity (48).

The data presented here on *Ft* XJPE highlights the importance of the often-overlooked impact of very long saturated fatty acids on the structure and regulation of biological membranes. Within the context of *Ft* pathogenicity, this study raises interesting questions. For example, with regard to the bacterial membrane itself it is unclear how high proportions of these very long–chain asymmetric lipids supported in *Ft* membranes without gel formation. The lower density in the membrane core induced by lipids with high tail asymmetry could impact more complex processes involving the membrane such as the permeation of small molecules. The effect of highly tail-asymmetric lipids on membrane order and fusion also needs further experiential and computational investigation, especially under realistic membrane compositions and with different membrane asymmetry. Finally, it is possible that the presence of highly tail-asymmetric phospholipids is critical for interaction and infection of host cells by aiding fusion or destabilizing the host membrane.

## Data availability

All simulation parameter files, setup files, and examples of equilibrated structures are available for download from https://bbs.llnl.gov/data.html.

## Supplemental data

Supporting information containing additional details of SAXS experiments and analysis, table of adjustable SAXS model parameters, lists of all simulations performed, results from control MD simulations, and additional analysis of MD simulations can be found online at https://doi.org/10.1016/j.jlr.2025.100797. CHARMM36 and Martini 3 topology files for XJPE, representative structure files of XJPE:DOPC, XJPE:DXPC and XJPE:POPC membranes can be found online at https://bbs.llnl.gov/data.html.

## Conflict of interest

The authors declare that they have no conflicts of interest with the contents of this article.
