# Supplementary material for: Highly tail-asymmetric lipids interdigitate and cause bidirectional ordering
Source: J Lipid Res. 2025 Apr 4;66(5):100797. doi: 10.1016/j.jlr.2025.100797 (PMC12135366; doi:10.1016/j.jlr.2025.100797)
Supplement: Supplemental data [file mmc1.docx]

Supporting Information for

Highly tail-asymmetric lipids interdigitate and cause bidirectional ordering

Tugba N. Ozturk, Thomas J. Ferron, Wei He, Benjamin Schwarz, Thomas M. Weiss, Nicholas O. Fischer, Amy Rasley, Timothy S. Carpenter, Catherine M. Bosio, Helgi I. Ingólfsson

**Additional details for All Atomistic (AA) and Coarse Grained (CG) Molecular Dynamics (MD) Simulation Analyses**

The total electron density profiles (shown in Figures 1C and S2C) and the number densities (presented in Figures 2B, 5D, S3B and S8D) were calculated with the GROMACS *density* tool [1]. Membrane thickness was estimated as the distance between two peaks in the total electron density profile of each membrane. Area per lipid (APL) was calculated as $\left( \left\langle L_{x} \right\rangle\left\langle L_{y} \right\rangle\right)/N$, where $\left\langle L_{x} \right\rangle$ and $\left\langle L_{y} \right\rangle$are the box dimensions along *x* and *y* axes averaged over the simulation trajectory and *N* is the number of lipids in a single leaflet. The area compressibility modulus, K_A_, was estimated as $k_{B}T \left\langle A \right\rangle/\left( N\left\langle\left( A-A_{0} \right)^{2} \right\rangle\right)$ [2]. Here, $k_{B}T$ is the Boltzmann constant multiplied with simulation temperature in Kelvin. A and A_0_ represent the instantaneous and the equilibrium areas of the simulation box on the *xy* plane. The lateral diffusion coefficients of lipids were calculated from the mean square displacement of the PO4 beads (or the P atoms) using the GROMACS *msd* tool [2]. The order parameter, P_2_, and the lipid enrichment/depletion indices were calculated using Lipyphilic [3]. The cut-off distance for lipid neighbor counts was set to 1.5 nm. Interleaflet contacts were calculated, with a distance cutoff of 0.6 nm, over a two dimensional grid on the *xy* plane in which the individual lipids were mapped using their GL1 GL2 (or C21 C31 atoms) [4]. The lateral pressure profiles were computed with GROMACS LS version 4.5.5 using the last 1 µs (10000 frames) of a 11 µs-long production run [5, 6]. During these calculations, covariant central force decomposition method was employed. The spacing for the pressure grid was set to 0.1 nm. The cutoff distances for electrostatic and Lennard-Jones interactions were set to 2.2 and 1.2 nm, respectively. The resulting profiles were then smoothened using a gaussian kernel with a standard deviation of 2 in the units of grid spacing. All snapshots were generated with VMD [7] and CG lipid representations were done with the *cg_bonds_v5.tcl* script [8].


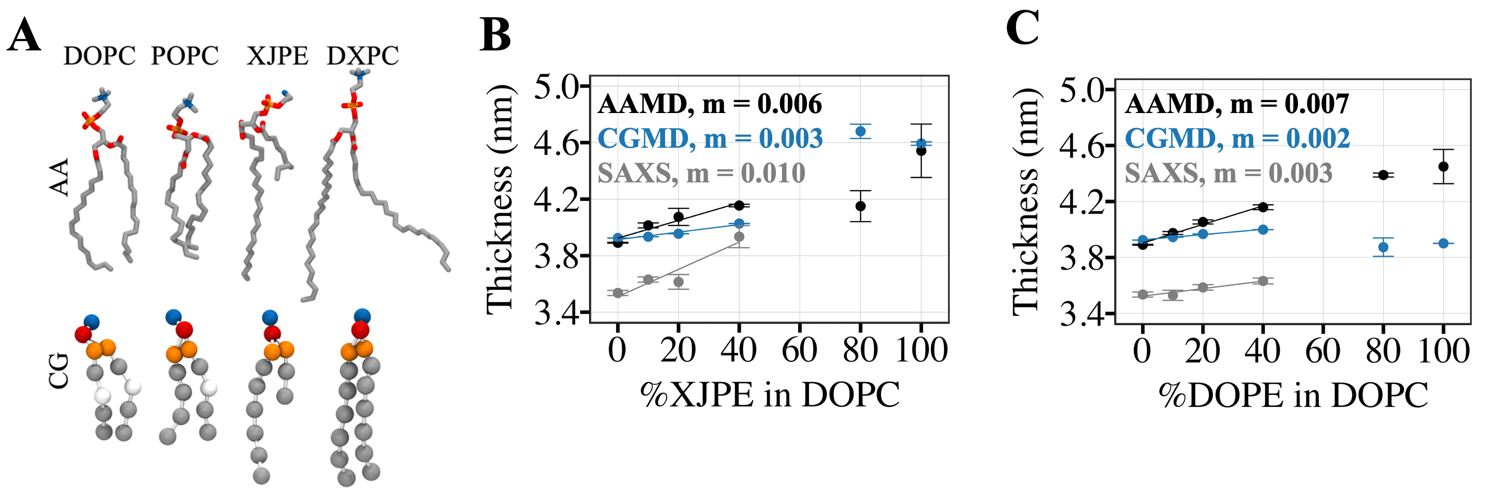


**Figure S1.** **(A)** All-atomistic (AA; top panel) and coarse-grained (CG; bottom panel) structural models of all the lipids studied in this work: DOPC (PC di18:1), POPC (PC 16:0/18:1), XJPE (PE 24:0/10:0), and DXPC (PC di24:0). Hydrogen atoms in the AA structural models were not shown for simplicity. The carbon, nitrogen, oxygen and phosphate atoms are represented as gray, blue, red and orange spheres, respectively. Similarly, PO4 beads in the CG models are colored in red, NC3/NH3 beads in blue, GL1 and GL2 beads in orange, C beads in gray, and finally D beads in white. The membrane thickness values estimated using AA MD simulations (black), CG MD simulations (blue) and SAXS scattering data (gray) were plotted against the percentage of **(B)** XJPE or **(C)** DOPE lipids in DOPC membranes. Panels B and C show larger versions of Figure 2C and Figure S3C with the addition of solid lines representing represent the linear fit for each data set only for membrane compositions correspond to fluid XJPE:DOPC membranes and the slope (m) for each fit is reported and fit R^2^ are between 0.86 and 0.99. The data show that there is small increase in membrane thickness until the phase separation occurs.

**Table S1.** The list of CG MD simulations performed in the study.

| Nr. | **Membrane composition**  **(% Lipid)** | **N_sim_** $\boldsymbol{\times}$ **t_sim_** | **Temperature** | **Force**  **Field** |
| --- | --- | --- | --- | --- |
| 1 | 100 POPE | 10 $\times$ 10 µs | 310, 315, 328 K | M3 |
| 2 | 100 XJPE | 10 $\times$ 10 µs | 310, 315, 328 K | M3 |
| 4 | 100 DXPC | 10 $\times$ 10 µs | 310, 315, 328 K | M3 |
| 5 | 90:10 POPE:DXPC | 10 $\times$ 10 µs | 310, 315, 328 K | M3 |
| 6 | 80:20 POPE:DXPC | 10 $\times$ 10 µs | 310, 315, 328 K | M3 |
| 7 | 90:10 XJPE:DXPC | 10 $\times$ 10 µs | 310, 315, 328 K | M3 |
| 8 | 80:20 XJPE:DXPC | 10 $\times$ 10 µs | 310, 315, 328 K | M3 |
| 11 | 100 POPC | 10 $\times$ 10 µs | 310 K | M3 |
| 12 | 10:90 XJPE:POPC | 10 $\times$ 10 µs | 310 K | M3 |
| 13 | 20:80 XJPE:POPC | 10 $\times$ 10 µs | 310 K | M3 |
| 14 | 30:70 XJPE:POPC | 10 $\times$ 10 µs | 310 K | M3 |
| 15 | 40:60 XJPE:POPC | 10 $\times$ 10 µs | 310 K | M3 |
| 16 | 50:50 XJPE:POPC | 10 $\times$ 10 µs | 310 K | M3 |
| 17 | 60:40 XJPE:POPC | 10 $\times$ 10 µs | 310 K | M3 |
| 18 | 70:30 XJPE:POPC | 10 $\times$ 10 µs | 310 K | M3 |
| 19 | 80:20 XJPE:POPC | 10 $\times$ 10 µs | 310 K | M3 |
| 20 | 90:10 XJPE:POPC | 10 $\times$ 10 µs | 310 K | M3 |
| 21 | 10:90 XJPE:POPC / 100 POPC* | 10 $\times$ 10 µs | 310 K | M3 |
| 22 | 20:80 XJPE:POPC / 100 POPC* | 10 $\times$ 10 µs | 310 K | M3 |
| 23 | 30:70 XJPE:POPC / 100 POPC* | 10 $\times$ 10 µs | 310 K | M3 |
| 24 | 40:60 XJPE:POPC / 100 POPC* | 10 $\times$ 10 µs | 310 K | M3 |
| 25 | 50:50 XJPE:POPC / 100 POPC* | 10 $\times$ 10 µs | 310 K | M3 |
| 26 | 60:40 XJPE:POPC / 100 POPC* | 10 $\times$ 10 µs | 310 K | M3 |
| 27 | 70:30 XJPE:POPC / 100 POPC* | 10 $\times$ 10 µs | 310 K | M3 |
| 28 | 80:20 XJPE:POPC / 100 POPC* | 10 $\times$ 10 µs | 310 K | M3 |
| 29 | 90:10 XJPE:POPC / 100 POPC* | 10 $\times$ 10 µs | 310 K | M3 |
| 30 | 100 XJPE /100 POPC* | 10 $\times$ 10 µs | 310 K | M3 |
| 31 | 100 DOPC | 4 $\times$ 10 µs | 298 K | M3 |
| 32 | 10:90 XJPE:DOPC | 4 $\times$ 10 µs | 298 K | M3 |
| 33 | 20:80 XJPE:DOPC | 4 $\times$ 10 µs | 298 K | M3 |
| 34 | 40:60 XJPE:DOPC | 4 $\times$ 10 µs | 298 K | M3 |
| 35 | 80:20 XJPE:DOPC | 4 $\times$ 10 µs | 298 K | M3 |
| 36 | 100 XJPE | 4 $\times$ 10 µs | 298 K | M3 |
| 37 | 10:90 DOPE:DOPC | 4 $\times$ 10 µs | 298 K | M3 |
| 38 | 20:80 DOPE:DOPC | 4 $\times$ 10 µs | 298 K | M3 |
| 39 | 40:60 DOPE:DOPC | 4 $\times$ 10 µs | 298 K | M3 |
| 40 | 80:20 DOPE:DOPC | 4 $\times$ 10 µs | 298 K | M3 |
| 41 | 100 DOPE | 4 $\times$ 10 µs | 298 K | M3 |
| 42 | 100 DOPC | 4 $\times$ 10 µs | 310 K | M3 |
| 43 | 10:90 XJPE:DOPC | 4 $\times$ 10 µs | 310 K | M3 |
| 44 | 20:80 XJPE:DOPC | 4 $\times$ 10 µs | 310 K | M3 |
| 45 | 40:60 XJPE:DOPC | 4 $\times$ 10 µs | 310 K | M3 |
| 46 | 80:20 XJPE:DOPC | 4 $\times$ 10 µs | 310 K | M3 |
| 47 | 10:90 XJPE:DOPC / 100 DOPC* | 4 $\times$ 10 µs | 310 K | M3 |
| 48 | 20:80 XJPE:DOPC / 100 DOPC* | 4 $\times$ 10 µs | 310 K | M3 |
| 49 | 40:60 XJPE:DOPC / 100 DOPC* | 4 $\times$ 10 µs | 310 K | M3 |
| 50 | 80:20 XJPE:DOPC / 100 DOPC* | 4 $\times$ 10 µs | 310 K | M3 |
| 51 | 100 XJPE / 100 DOPC* | 4 $\times$ 10 µs | 310 K | M3 |

*Systems contain the XJPE lipids only on the outer leaflet. For these systems, the compositions of the outer leaflet and inner leaflet are separated with a slash.

**Table S2.** The list of AA MD simulations performed in the study.

|  | **Membrane composition**  **Percentage Lipid** | **N_sim_** $\boldsymbol{\times}$ **t_sim_** | **Temperature** | **Force**  **Field** |
| --- | --- | --- | --- | --- |
| 1 | 100 DOPC | 4 $\times$ 350 ns | 298 K | C36 |
| 2 | 10:90 XJPE:DOPC | 4 $\times$ 350 ns | 298 K | C36 |
| 3 | 20:80 XJPE:DOPC | 4 $\times$ 350 ns | 298 K | C36 |
| 4 | 40:60 XJPE:DOPC | 4 $\times$ 350 ns | 298 K | C36 |
| 5 | 80:20 XJPE:DOPC | 4 $\times$ 350 ns | 298 K | C36 |
| 6 | 100 XJPE | 4 $\times$ 350 ns | 298 K | C36 |
| 7 | 10:90 DOPE:DOPC | 4 $\times$ 350 ns | 298 K | C36 |
| 8 | 20:80 DOPE:DOPC | 4 $\times$ 350 ns | 298 K | C36 |
| 9 | 40:60 DOPE:DOPC | 4 $\times$ 350 ns | 298 K | C36 |
| 10 | 80:20 DOPE:DOPC | 4 $\times$ 350 ns | 298 K | C36 |
| 11 | 100 DOPE | 4 $\times$ 350 ns | 298 K | C36 |


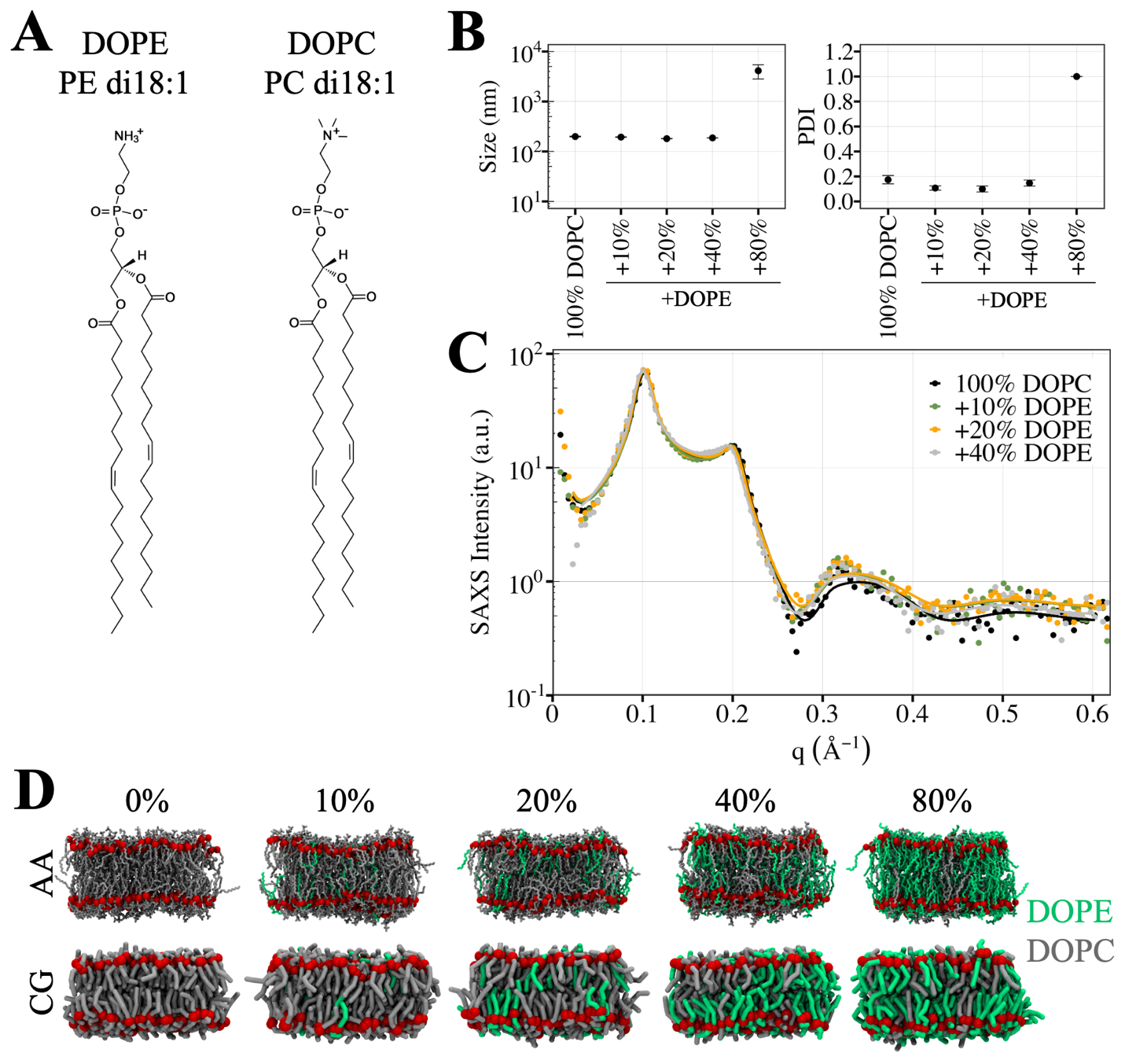


**Figure S2.** Phase behaviors of DOPE:DOPC membranes. **(A)** The structures of DOPE and DOPC. **(B)** Average size (left panel) and polydispersity index (PDI; right panel) values for DOPC liposomes containing 0%, 10%, 20%, 40% and 80% DOPE. The data points are averaged over three independent replicas and the standard deviation around the mean are shown as error bars. **(C)** SAXS intensity profiles of DOPC liposomes containing 0%, 10%, 20% and 40% DOPE lipids. Raw and fitted data points are represented as filled circles and solid lines, respectively. **(D)** The snapshots of DOPE:DOPC membranes at the end of 350 ns-long AA and 10 µs-long CG MD simulations. DOPE and DOPC lipids are colored orange and gray, respectively. Red spheres show the glycerol linker, C21 and C31 atoms in the AA systems and GL1 and GL2 beads in the CG systems.


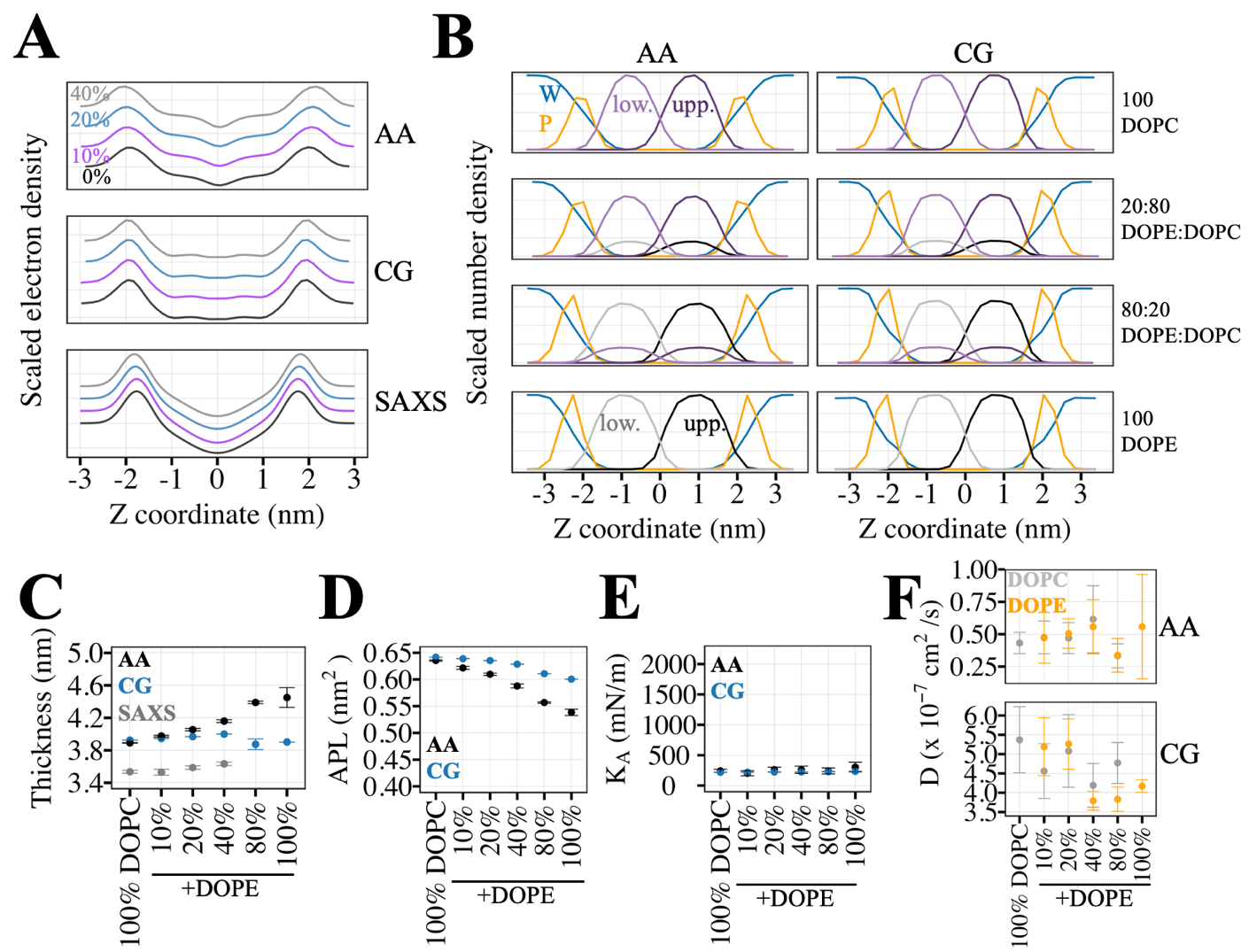


**Figure S3.** Properties of DOPE:DOPC membranes. **(A)** Shown are the total electron density profiles of DOPE:DOPC membranes along the membrane normal. The top, middle and bottom panels are the profiles generated using AA, CG MD simulation data and SAXS data, respectively. The colored-lines represent the data for different membrane compositions: black – 0:100 DOPE:DOPC, purple – 10:90 DOPE:DOPC, blue – 20:80 DOPE:DOPC and gray – 40:60 DOPE:DOPC. **(B)** The number density profiles of water (blue), phosphates (orange), DOPE tails (black – upper leaflet; gray – lower leaflet) and DOPC tails (purple – upper leaflet; light purple – lower leaflet) were computed from the AA (left) and CG (right) MD simulations. The mean values of the **(C)** membrane thickness, **(D)** area per lipid (APL), **(E)** area compressibility modulus (K_A_) and **(F)** lateral diffusion coefficients (D) were computed for each DOPE:DOPC membrane composition and averaged over all repeats. The standard deviation around the mean was calculated using 4 independent AA or 10 independent CG MD simulations for each membrane composition. The membrane thickness was estimated as the distance between two peaks as shown in Panel **A**.


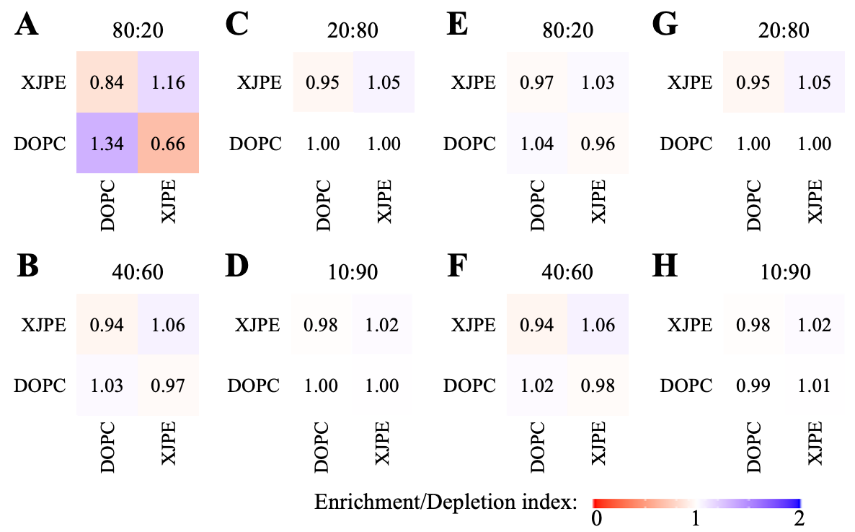


**Figure S4.** XJPE lipids mix well within liquid DOPC membranes. Enrichment/depletion indices of XJPE:DOPC membranes at **(A)** 80:20, **(B)** 40:60, **(C)** 20:80 and **(D)** 10:90 compositions, calculated from 4 independent 10 μs-long CG MD simulations at 298 K. Note, in all CG MD simulations of 80:20 XJPE:DOPC membranes, larger parts of the membranes have entered gel phase. The enrichment/depletion indices of **(E)** 80:20, **(F)** 40:60, **(G)** 20:80 and **(H)** 10:90 XJPE:DOPC membranes simulated at 310 K are also shown. The enrichment/depletion indices can take any value between 0 to 2; the smaller (red) the index is, the more depleted the lipid pair is and the more positive (blue) the index is, the more enriched the lipid pair is. The indices are averaged for both leaflets and are calculated from the counts of each lipid species around 1.5 nm of each lipid using LiPyphilic’s neighbors module [3].


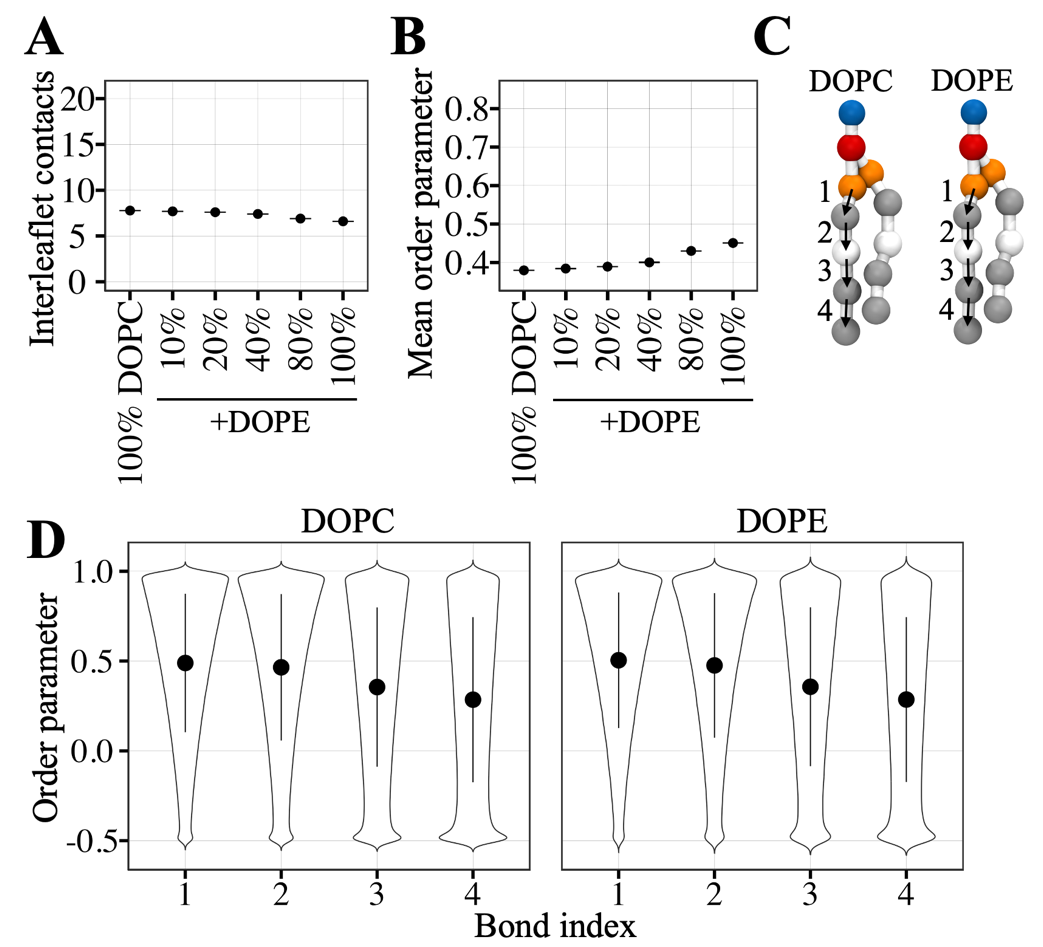


**Figure S5.** Interleaflet contacts and lipid ordering in DOPE:DOPC membranes. **(A)** Average interleaflet contacts for DOPE:DOPC membranes at different ratios. Error bars show the standard deviation around the mean computed over the course of 4 independent CG MD simulations of each membrane composition. **(B)** Mean order parameter is calculated as an average over all tail bonds of lipids through independent CG MD simulations. **(C)** Tail bond indices are shown for the *sn1* tails of DOPC (left) and DOPE (right) lipids. **(D)** The distributions of the P_2_ order parameter of each bond shown in Panel **C** are given for DOPC (left) and DOPE (right) lipids using the CG simulations of 20:80 DOPE:DOPC membranes at 298 K. The solid circle and lines represent mean and mean $\pm$ standard deviation, respectively.


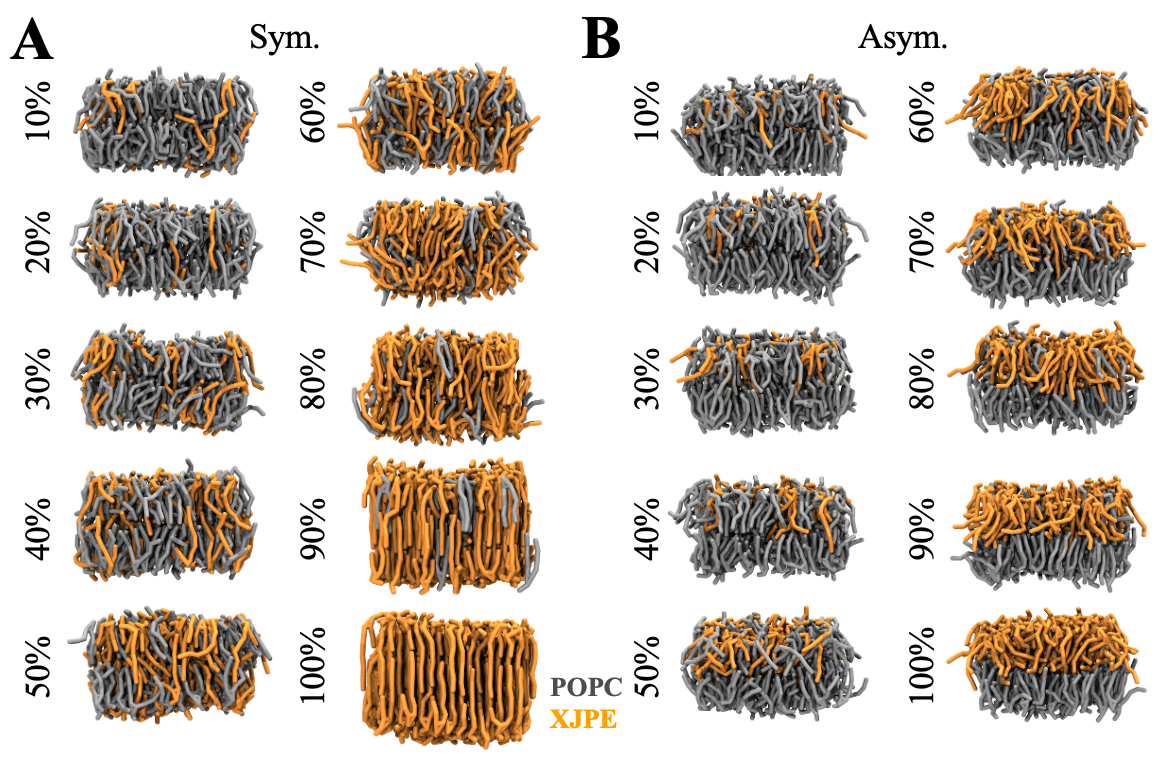


**Figure S6.** Configurations of XJPE:POPC membranes at the end of 10 μs-long simulations performed at 310 K. Simulation snapshots showing a set of XJPE:POPC membranes that include 10-100% XJPE lipids in both leaflets of the membrane **(A)** or where the XJPE lipids are only added to the outer leaflet of the membranes **(B)**. XJPE and POPC lipids are represented as licorice and colored orange and gray, respectively. The percentage of XJPE lipids in each membrane is given in the left side of the snapshot.


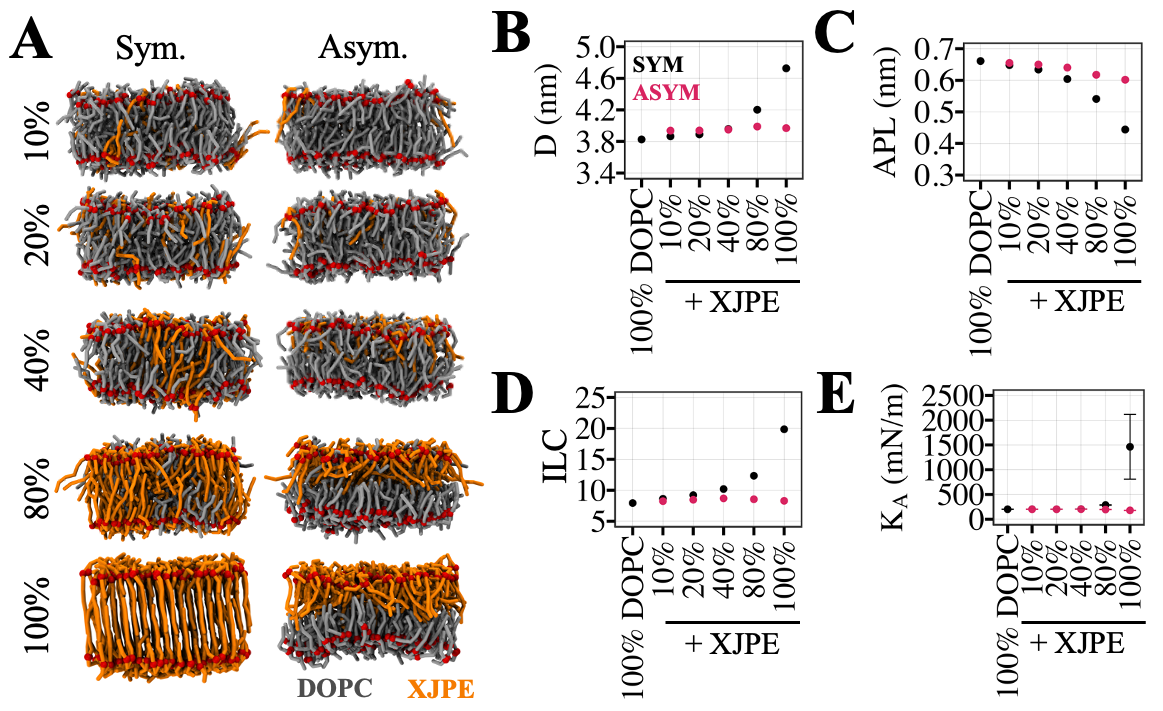


**Figure S7.** Properties of XJPE:DOPC membranes. **(A)** Snapshots of XJPE:DOPC membranes are shown at different compositions where XJPE is added either symmetrically, into both leaflets (left column), or asymmetrically, only into the outer leaflet (right column). The mean values of **(B)** membrane thickness, **(C)** area per lipid (APL), **(D)** interleaflet contacts (ILC) and **(E)** area compressibility modulus (K_A_) are computed over 4 independent CG MD simulations and shown without and with increasing concentration of XJPE lipids in the membranes. The black and pink data points represent the symmetric and asymmetric membrane mixtures, respectively. In Panel **B-D**, the error bars are not shown as they are smaller than 0.1 nm, 0.005 nm^2^ and 0.8, respectively. In Panel **E**, the shown error bars represent the standard deviation around the mean.

**
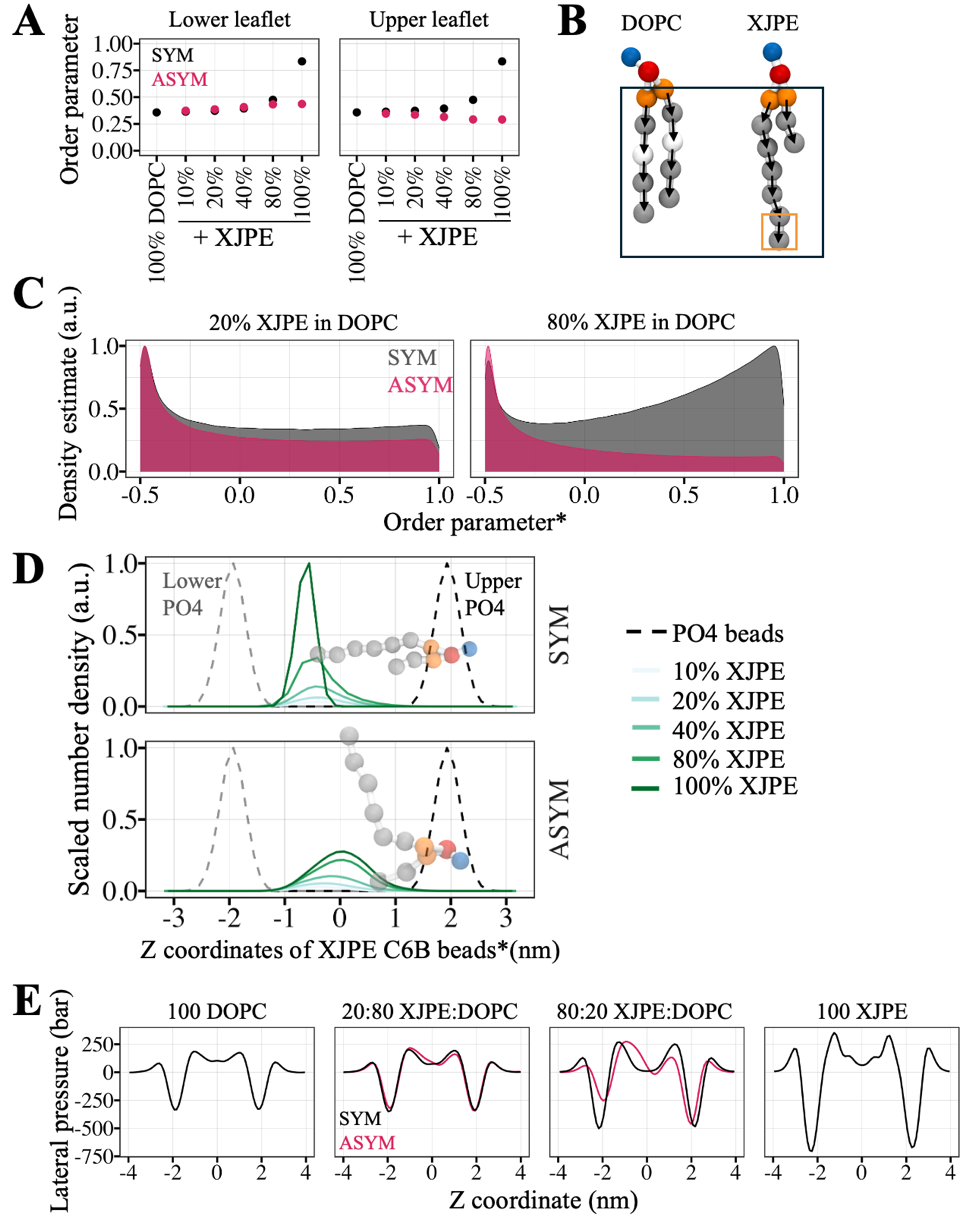
**

**Figure** **S8.** Lipid ordering in XJPE:DOPC membranes. **(A)** Mean order parameter is plotted against the membrane composition. The data for symmetric and asymmetric XJPE:DOPC membranes are shown in black and pink, respectively. The mean order parameter averaged for all the lipids was computed for those in the lower (left) and upper (right) leaflets separately. **(B)** The ball and stick representation of CG DOPC (left) and XJPE (right) lipids. The black frame shows the tail bonds used to calculate the mean order parameters presented in Panel **A** while the orange frame highlights the last tail bond in the XJPE’s sn1 tail that is used to calculate the order parameter distribution shown in Panel **C**. **(C)** The scaled density of order parameter was calculated for the last bond of XJPE’s sn1 tail using the CG MD simulations of 20:80 XJPE:DOPC and 80:20 XJPE:DOPC at 310 K. The data for symmetric (black) and asymmetric (pink) XJPE:DOPC membranes are shown with 20% transparency. **(D)** The scaled number density of the last tail bead was plotted for the sn1 tail of XJPE lipids in the upper leaflets of symmetric (top) and asymmetric (bottom) XJPE:DOPC membranes. A representative XJPE lipid configuration is scaled properly for the calculated density profiles and are shown transparently on the graphs. The solid lines represent the number density of tail beads and are colored from light to dark green for 10:90, 20:80, 40:60, 80:20, and 100:0 XJPE:DOPC as labelled whereas the dashed lines represent the phosphate beads in the lower (gray) and upper (black) leaflets from the CG MD simulations of 20:80 XJPE:DOPC membranes. In Panels **C** and **D**, the star symbols in the x axis label highlight that the shown data is computed only for the XJPE lipids in the upper leaflet of the membranes. **(E)** Lateral stress profiles for 0:100, 20:80, 80:20 and 100:0 XJPE:DOPC membranes, given from left to right, are plotted against the membrane normal and are colored black and pink for the symmetric and asymmetric membranes, respectively.

**
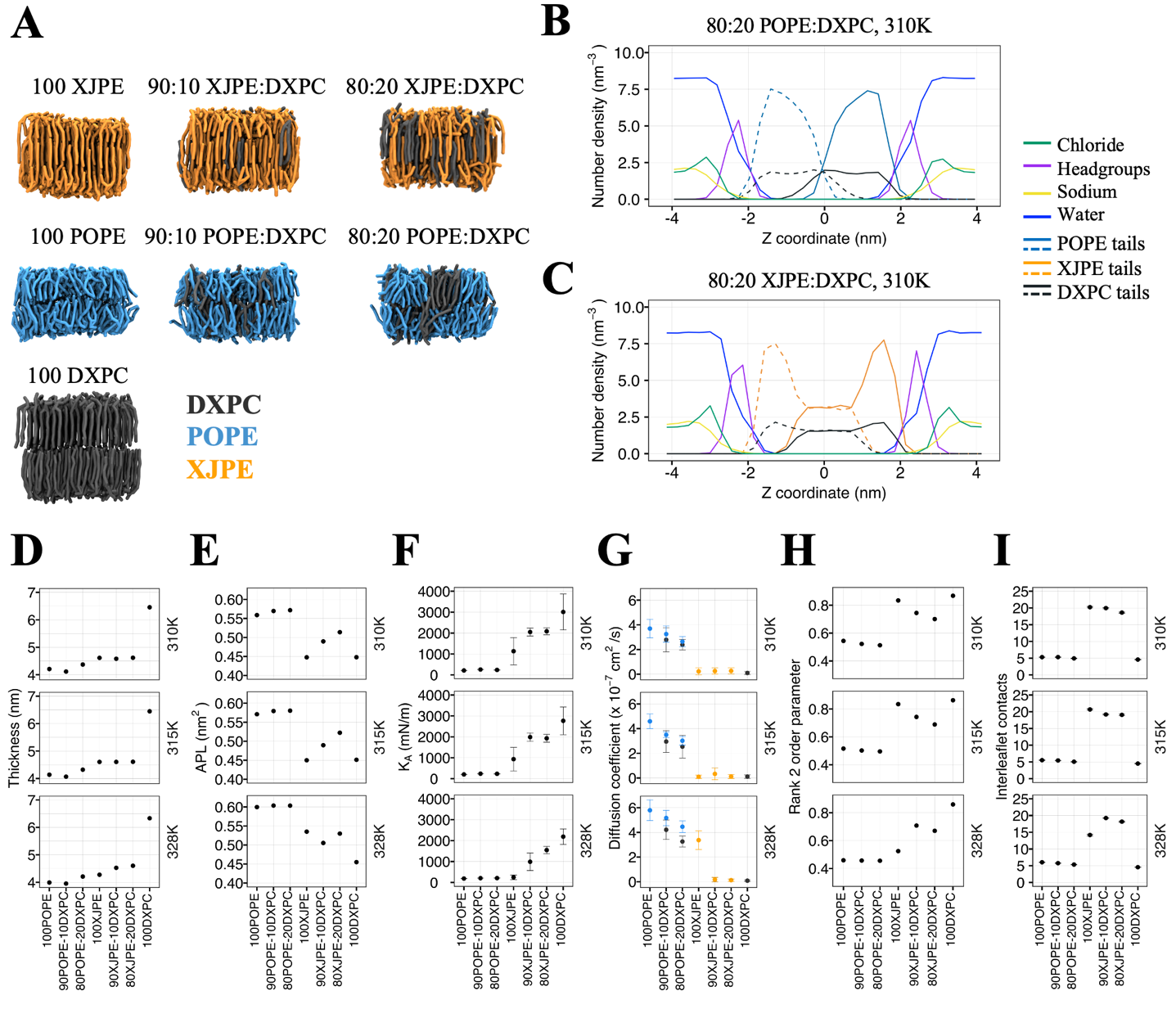
**

**Figure S9.** Impact of XJPE and POPE in DXPC membranes. **(A)** The snapshots of XJPE:DXPC and POPE:DXPC (control) membranes at 310K are generated using the last frame of representative CG MD simulation trajectories. The number density profiles for water, salt ions, head groups and tails in the upper (solid lines) and lower (dashed lines) leaflets are computed along the membrane normal and color-coded for **(B)** 80:20 POPE:DXPC and **(C)** 80:20 XJPE:DXPC membranes at 310 K. Membrane properties were plotted for each membrane composition at 310 K (top panels), 315 K (middle panels) and 328 K (bottom panels): **(D)** membrane thickness, **(E)** APL, **(F)** K_A_, **(G)** lateral diffusion coefficient, **(H)** average order parameter and **(I)** number of interleaflet contacts are shown for XJPE:DXPC and POPE:DXPC membranes using the CG MD simulation trajectories performed at 310, 315 and 328 K. Lipids and their specific properties are colored in orange (XJPE), blue (POPE) and gray (DXPC). Note that the error bars are calculated as the standard deviation around the mean computed using 10 independent CG MD simulations. In Panels **B**, **C**, **F**, and **G** the error bars were not shown as they were smaller than 0.16 nm, 0.003 nm^2^, 0.01, and 0.38, respectively.

**Additional details for Small-Angle X-ray Scattering (SAXS) Experiments and Analysis**

The liposome morphology is modelled as a large hollow sphere with the electron density of the shell described by three gaussian functions. The two outer gaussians represent volume probability distributions of the headgroups in either leaflet (with a greater electron density than water) while the inner gaussian gives the location of the lowest electron density (negative when compared to water) that predominately consists of the alkyl chains and terminal methyl groups of both leaflets. The best-fit results are presented as solid lines in Figure 1C of the paper, and the resulting electron density profiles are given in Figure 2A. In order to properly capture the shape of the SAXS profiles, we were required to allow for variable headgroup positioning between the two leaflets and for the region of the lowest electron density to be offset from the bilayer midplane. Either effect alone would not successfully model the data, neither would a perfectly symmetric electron density profile.

Variable headgroup positioning could result from either a difference in curvature between the inner and outer leaflets or a variable lipid composition. Lipids with PE headgroups have been shown to preferentially organize at the inner leaflet in a mammalian-like bilayer which may have occurred spontaneously during assembly or dynamically prior to SAXS measurements via lipid flip-flop [9]. Furthermore, the asymmetry in the electron density profile within the hydrophobic region suggests some offset of the center-of-mass of methyl groups and the most likely candidate for this phenomenon, given our system, is from interdigitation. This would require one of the one of the leaflets be enriched with XJPE to have a concentration of chains on one half of the bilayer. A more sophisticated scattering model with a joint analysis of X-ray and neutron scattering may help resolve these outstanding questions [10]. Work is ongoing to improve the population of unilamellar XJPE-containing liposomes in our suspensions as well as purposefully fabricate compositionally asymmetric liposomes for more controlled study on chain-interdigitation.

**Scattering Model**

Scattering from a suspension of liposomes can be modeled as a product of a form factor, $F\left( q \right)$, that describes SAXS from large unilamellar vesicles (LUVs), and the structure factor, $S\left( q \right)$, accounting for interlamellar ordering (i.e. stacked bilayers) commonly found in multilamellar or nested vesicles (MLVs). The measured scattering intensity, $I\left( q \right)$, can be calculated with the relationship,

|  | $I\left( q \right)\propto\left\vert F\left( q \right) \right\vert^{2}S\left( q \right),$ | (1) |
| --- | --- | --- |

where $q$ is the momentum transfer vector defined as $q=4\pi\sin\left( 2\theta\right)/\lambda$, $\lambda$ is the X-ray wavelength and $\theta$ is the scattering angle. A proportionality relationship is used since we do not measure the scattering intensity on an absolute scale.

**Form Factor**

The radius of an LUV in this study is significantly greater than the bilayer thickness allowing us to apply the method of separable form-factors described by Pencer *et al.* [11] such that,

|  | $F\left( q \right)\approx F_{TS}\left( q \right)F_{M}\left( q \right),$ | (2) |
| --- | --- | --- |

where $F_{TS}\left( q \right)$ is the form factor of an infinitely thin shell and $F_{M}\left( q \right)$ is the form factor of a bilayer membrane. The q-range of our SAXS data precludes us from uniquely determining the size and polydispersity of the measured LUV population and instead, dynamic light scattering (DLS) measurements can provide us with that characterization. Therefore, we choose to simply Equation 2 by substituting the Lorentz correction, $1/q^{2}$, in place of $F_{TS}\left( q \right)$ as demonstrated previously [12]. The function $F_{M}\left( q \right)$ takes the form,

|  | $F_{M}\left( q \right)=\int_{z} \rho\left( z \right)\left[ \cos\left( qz \right)+i\sin\left( qz \right) \right]dz$ | (3) |
| --- | --- | --- |

where we assume that the LUV is radially symmetric and the membrane bilayer can be described by a one-dimensional scattering length density (SLD) profile, $\rho\left( z \right)$, where $z=0$is defined as the bilayer midplane. The integral in Equation 3 is over the full thickness of the bilayer membrane.

The function $\rho\left( z \right)$ is the primary means at which we describe the membrane morphology. Multiple strategies exist to accurately match $\rho\left( z \right)$ with a physical picture of the lamellar phase and in this study we choose to construct $\rho\left( z \right)$ by summing three gaussian functions,

|  | $\rho\left( z \right)=\sum_{i=1}^{3} \left( \rho_{i}-\rho_{0} \right)\exp\left( -\left( z-z_{i} \right)^{2}/2\sigma_{i}^{2} \right),$ | (4) |
| --- | --- | --- |

where $\rho_{i}$ is the magnitude of the SLD, $z_{i}$ is the position, and $\sigma_{i}$ is the width of the $i^{th}$ gaussian function, and $\rho_{0}$ is the SLD of the buffer solution. In this profile, the two outer gaussians represent the position of the headgroups while the middle gaussian function represents the interdigitation of the hydrocarbon chains and the terminal methyl group. Eq. 4 is capable of modeling both symmetric and asymmetric membrane bilayers depending on the relative position of the outer two gaussians compared to the center. Both types of membranes were used to model data in this study. An example bilayer profile is presented in Figure S9 for the special case of an asymmetric bilayer.


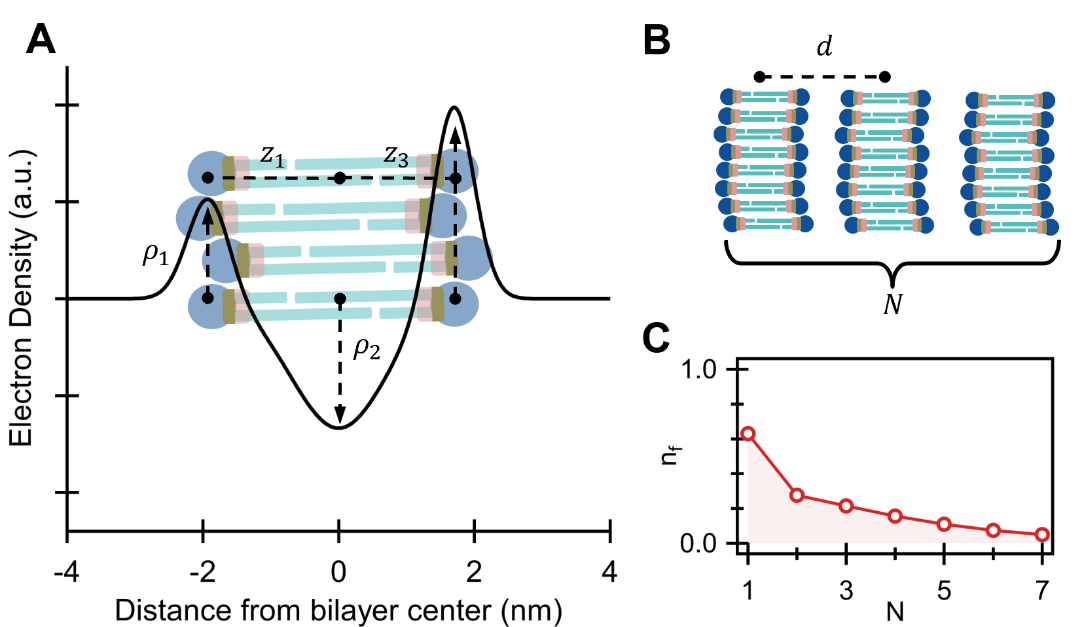


**Figure S10.** **(A)** Example electron density profile that demonstrates the meaning of each parameter used in the bilayer form-factor. **(B)** Cartoon depicting the structure factor found in multilamellar vesicles and what parameters are used to describe them. **(C)** Example function of $n_{f}$ for a suspension of DOPC liposomes considering particles with up to 7 bilayers.

**Structure Factor**

In the presence of MLV’s the addition of $S\left( q \right)$ into Eq. 1 accounts for interlamellar diffraction and can be described by Caillé theory according to,

|  | $S_{N}\left( q \right)=N+2\sum_{k=1}^{N-1} \left( N-k \right)\cos\left( kqd \right)\exp\left( -\left( dq/2\pi\right)^{2}\eta\left\{ \gamma+\ln\left( \pi k \right) \right\} \right),$ | (5) |
| --- | --- | --- |

where $N$ is the total number of bilayers in the MLV, $d$ is the interlamellar spacing, $\eta$ is the Caillé parameter (related to bending rigidity of the bilayer membrane), and $\gamma$ is Euler’s constant[13, 14]. Figure S9B shows a cartoon depicting the parameters used in the structure factor. To account for an MLV suspension with particles that contain varying numbers of bilayers, we compute an ensemble averaged structure factor, $\left\langle S\left( q \right) \right\rangle$, using a strategy developed by Scott et al.[15]. We begin with a distribution, $w_{N}\left( \lambda\right)$, that defines the probability of finding a vesicle in the suspension with $N$ bilayers,

|  | $w_{N}\left( \lambda\right)=\left\{ \begin{matrix} w_{1} & N=1 \\ \exp\left( -\lambda N \right) & 1<N\leq N_{max} \\ 0 & N>N_{max} \end{matrix}. \right.$ | (6) |
| --- | --- | --- |

where $N_{max}$ is the maximum number of bilayers considered in the suspension, $w_{1}$ is an empirical correction to more heavily weigh the probability of finding ULVs in the suspension and $\lambda$ is defined by the choice in $N_{max}$ at which point the distribution is truncated,

|  | $\lambda=\left( -\log\left( 1-p \right)/N_{max} \right),$ | (7) |
| --- | --- | --- |

where $p=0.999$ is the chosen probabilistic cutoff. The function $w_{N}\left( \lambda\right)$ can be reduced to a number fraction, $n_{f}$, by normalizing to $\sum_{N} w_{N}\left( \lambda\right)$ and an example function is given in Figure S9C. The resulting value of $\left\langle S\left( q \right) \right\rangle$ is then calculated using,

|  | $\left\langle S\left( q \right) \right\rangle={\sum_{N=1}^{N_{max}} S_{N}\left( q \right)w_{N}\left( \lambda\right)}/{\sum_{N=1}^{N_{max}} w_{N}\left( \lambda\right).}$ | (8) |
| --- | --- | --- |

**SAXS Data Modeling Strategy**

Scattering data were fit to a modified version of Eq. 1 given by,

|  | $I\left( q \right)=C/q^{2}\left\vert F_{M}\left( q \right) \right\vert^{2}\left\langle S\left( q \right) \right\rangle+b,$ | (9) |
| --- | --- | --- |

where $C$ is a global scale parameter, $b$ is an additive background, $F_{m}\left( q \right)$ is given by Eq. 3 and $\left\langle S\left( q \right) \right\rangle$ is given by Eq. 8. A list of all adjustable parameters to the model is given in Table S3. To improve the uniqueness of our fitting routine, we make the following assumptions about the structure. First, the gaussian functions describing the headgroup are given a fixed with of $\sigma=3 Å$ while the inner gaussian has a constant with of $\sigma=7 Å$. Secondly, since we are not calibrating the SAXS intensity to an absolute magnitude, we only consider the relative change in $\rho\left( z \right)$ throughout the SLD profile. Therefore, we set $\rho_{0}=0$, fix the magnitude of $\rho_{1}=1$ and only consider the relative magnitude of $\rho_{2}$ and $\rho_{3}$ compared to $\rho_{1}$. Lastly, for symmetric bilayer membranes, $\rho_{1}=\rho_{3}$ and $z_{1}=-z_{3}$. The model is then applied on up to eight different datasets that includes two solution concentrations, 2 and 5 mg/ml, and multiple duplicates. The full set of SAXS data, models, and extracted parameters can be found in Figures S11-S18.The reported values in the main text are taken from the mean result of each sample series.

**Table S3.** Adjustable parameters for SAXS model. Fixed values are empirically chosen during model optimization to reduce variable parameters. Small deviations about those values will result in nearly equivalent fits.

| **Symbol** | **Description** | **Status** |
| --- | --- | --- |
| $C$ | Arbitrary Scale Factor | Open Parameter |
| $b$ | Additive Background | Open Parameter |
| ***Form Factor*** | | |
| $z_{1}$ | Position of first Gaussian | Open Parameter |
| $\sigma_{1}$ | Width of first Gaussian | Held at 3 Å |
| $\rho_{1}$ | Height/SLD of first Gaussian | Held at 1 |
| $z_{2}$ | Position of second Gaussian | Held at $z=0$ |
| $\sigma_{2}$ | Width of second Gaussian | Held at 7 Å |
| $\rho_{2}$ | Height/SLD of second Gaussian | Open Parameter |
| $z_{3}$ | Position of third Gaussian | Open only for asymmetric profiles |
| $\sigma_{3}$ | Width of third Gaussian | Held at 3 Å |
| $\rho_{3}$ | Height/SLD of third Gaussian | Open only for asymmetric profiles |
| ***Structure Factor*** | | |
| $N_{max}$ | Max number of bilayers | Held at 7 |
| $w_{x}$ | Probability of vesicles with $x$ layers | Open Parameter |
| $\eta$ | Caillé parameter | Open parameter |
| $d$ | Interlamellar spacing | Open parameter |


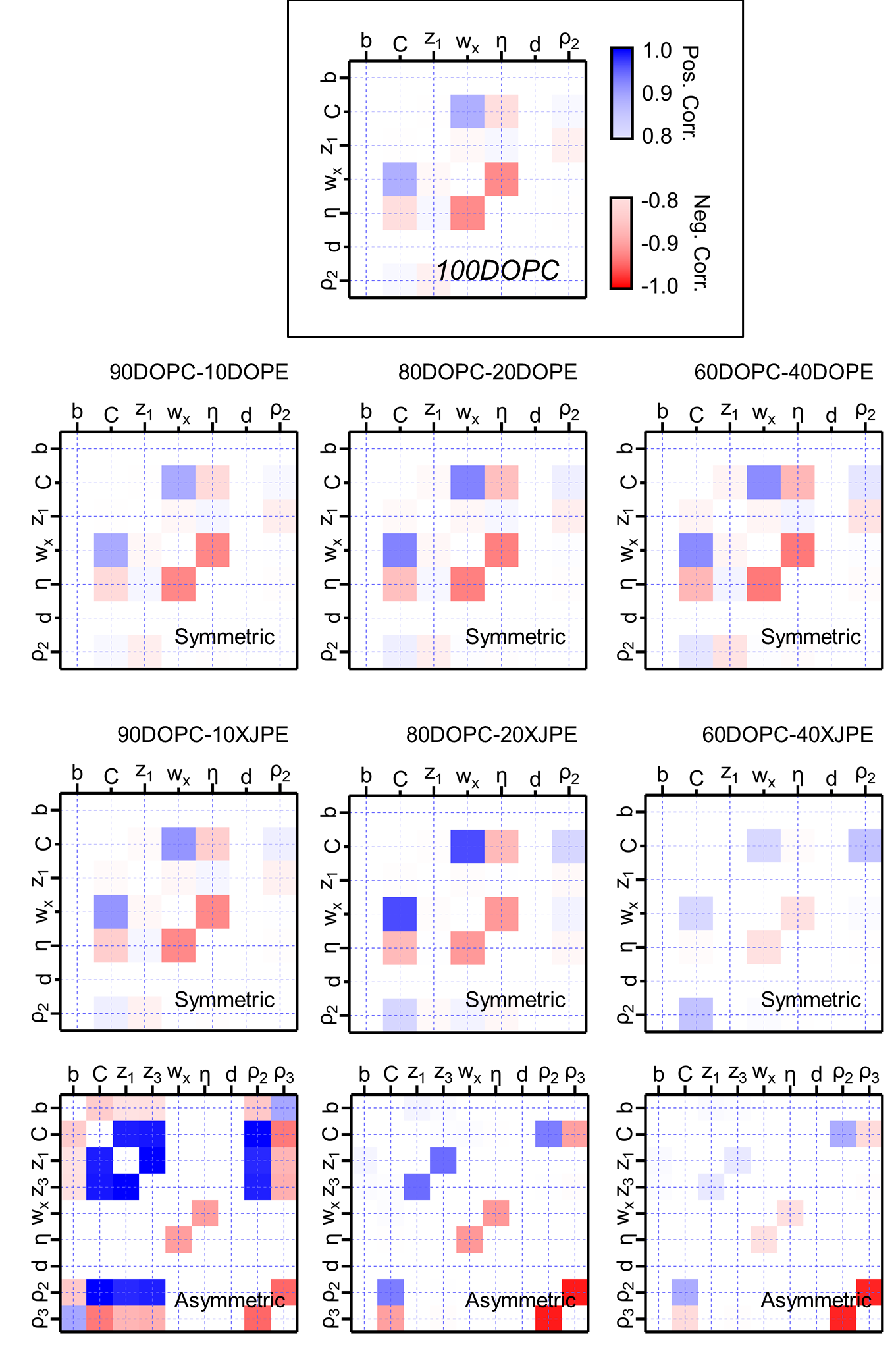


**Figure S11.** Complication of parameter correlation matrices of each model computed during least chi-squared optimization. Each matrix corresponds to a best-fit result for solution concentrations of 5 mg/ml. The indication of ‘Symmetric’ or ‘Asymmetric’ indicates whether the outer two Gaussian functions (corresponding to the position of opposing leaflet headgroups) are mirrors of each other. The ‘Asymmetric’ functions have a greater number of open parameters to account for that difference. We find some correlations during parameter fitting but no two parameters are perfectly correlated. The color scale reported for the 100% DOPC sample is used in all panels.


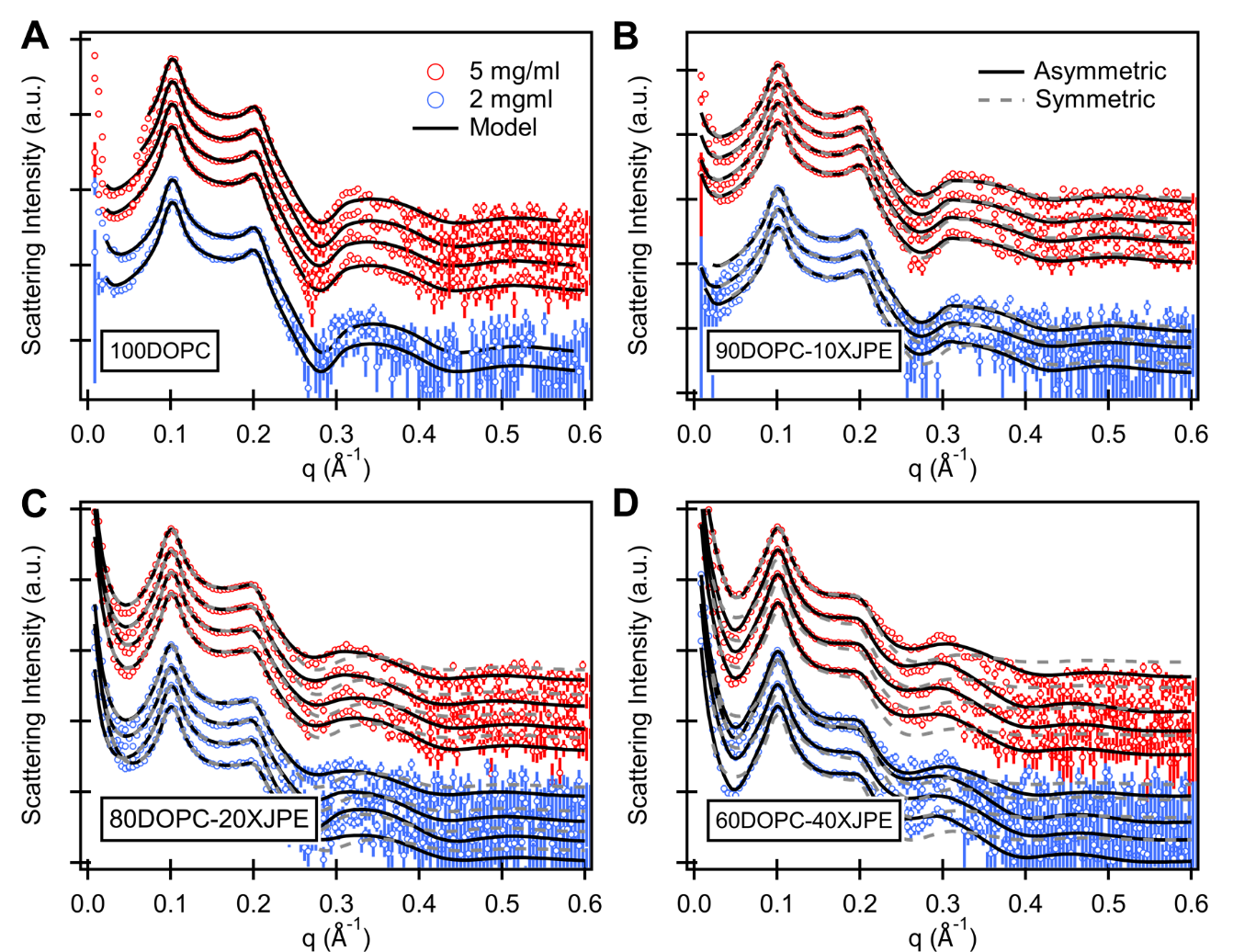


**Figure S12.** SAXS data and models for liposome blends that contain DOPC and **(A)** 0%, **(B)** 10%, **(C)** 20%, and **(D)** 40% XJPE. Each trace represents a measurement conducted from a different sample that was aliquoted and diluted from a bulk suspension. Data has been linearly offset for clarity. The marker and lineout legends in Panels **A** and **B** are used in all panels.


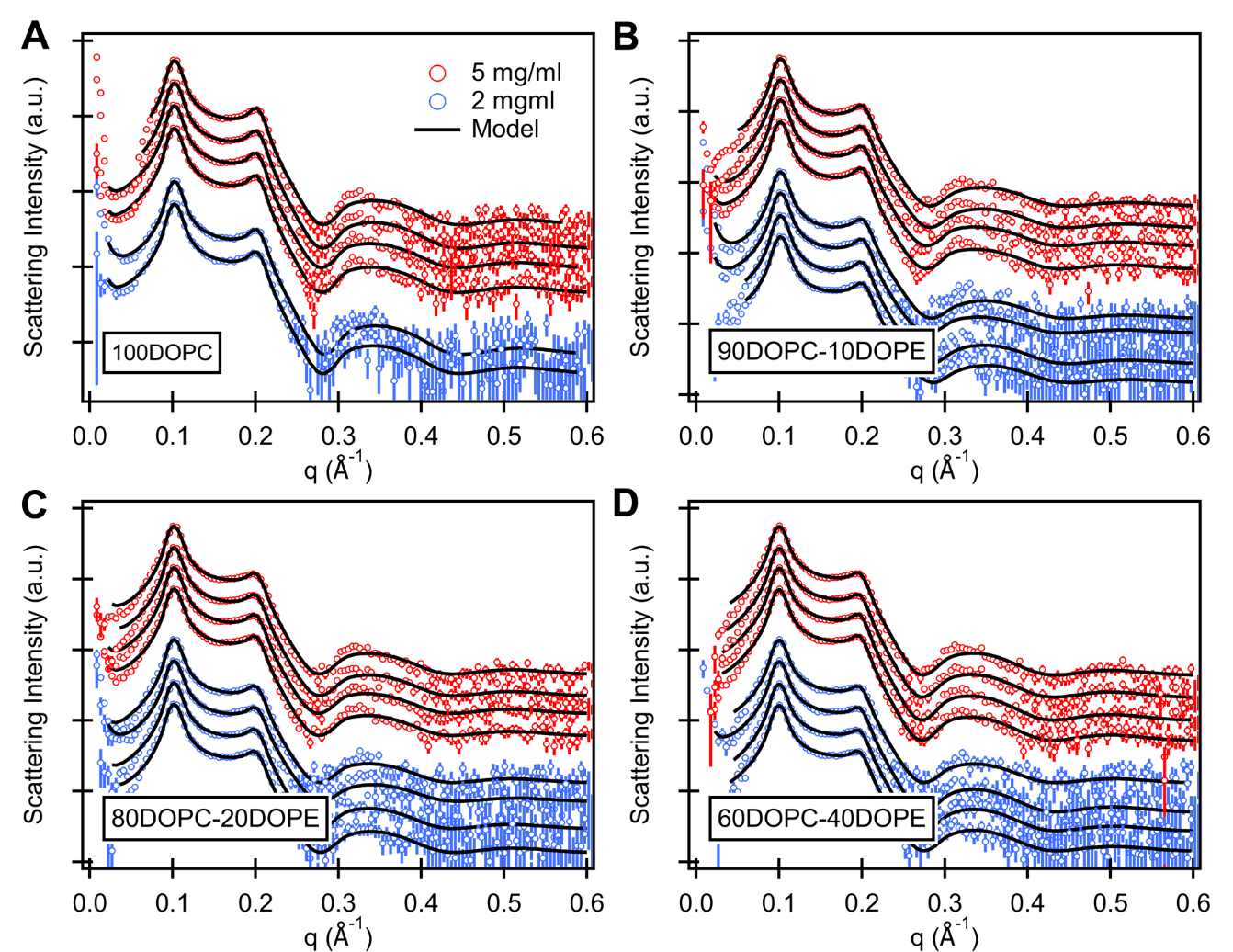


**Figure S13.** SAXS data and models for liposome blends that contain DOPC and **(A)** 0%, **(B)** 10%, **(C)** 20%, and **(D)** 40% DOPE. Each trace represents a measurement conducted from a different sample that was aliquoted and diluted from a bulk suspension. Data has been linearly offset for clarity. The marker legend in Panel **A** is used in all panels and the model is symmetric only.


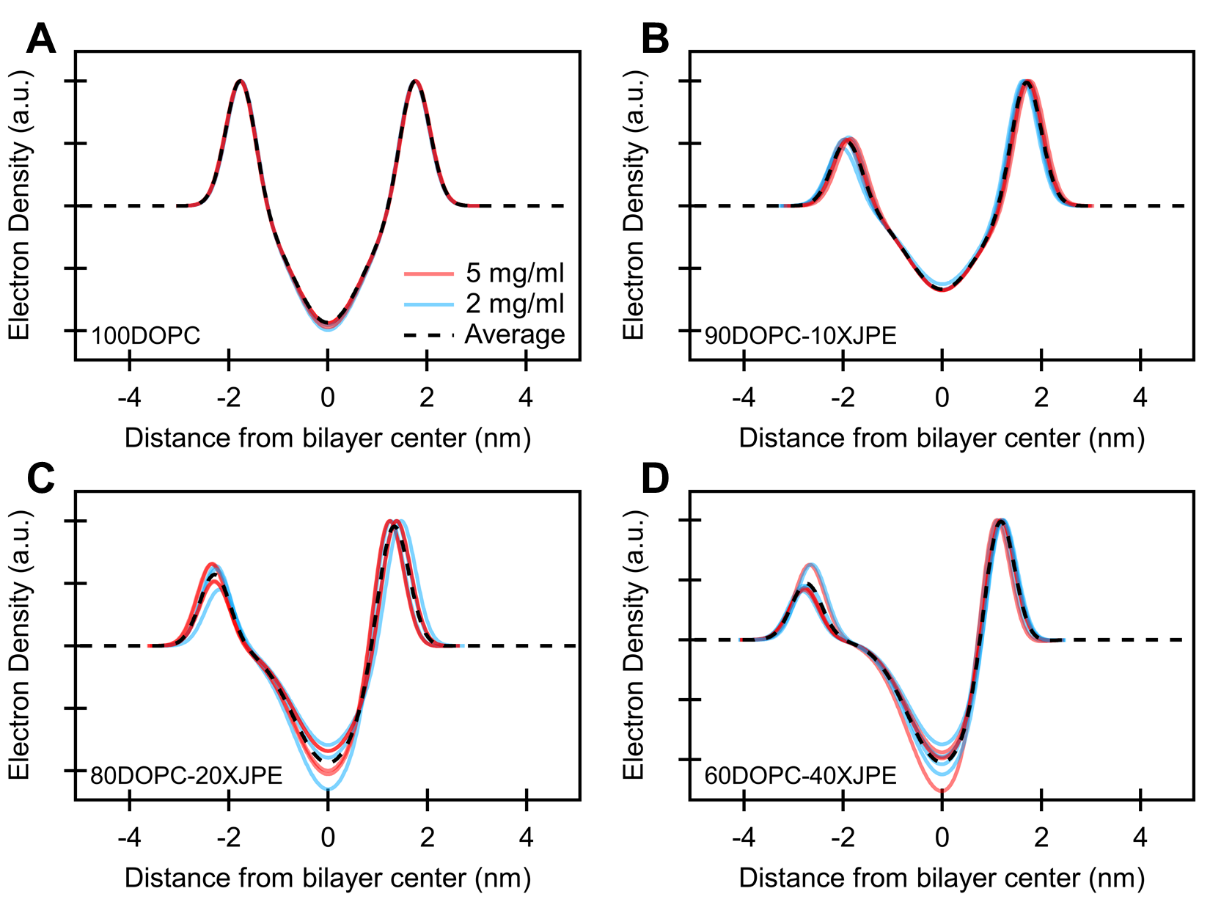


**Figure S14.** Asymmetric electron density profiles from scattering models displayed in Figure S11 for DOPC with **(A)** 0%, **(B)** 10%, **(C)** 20%, and **(D)** 40% XJPE. All profiles are overlayed to demonstrate differences between individual models. The marker legends in Panel **A** are used in all panels.


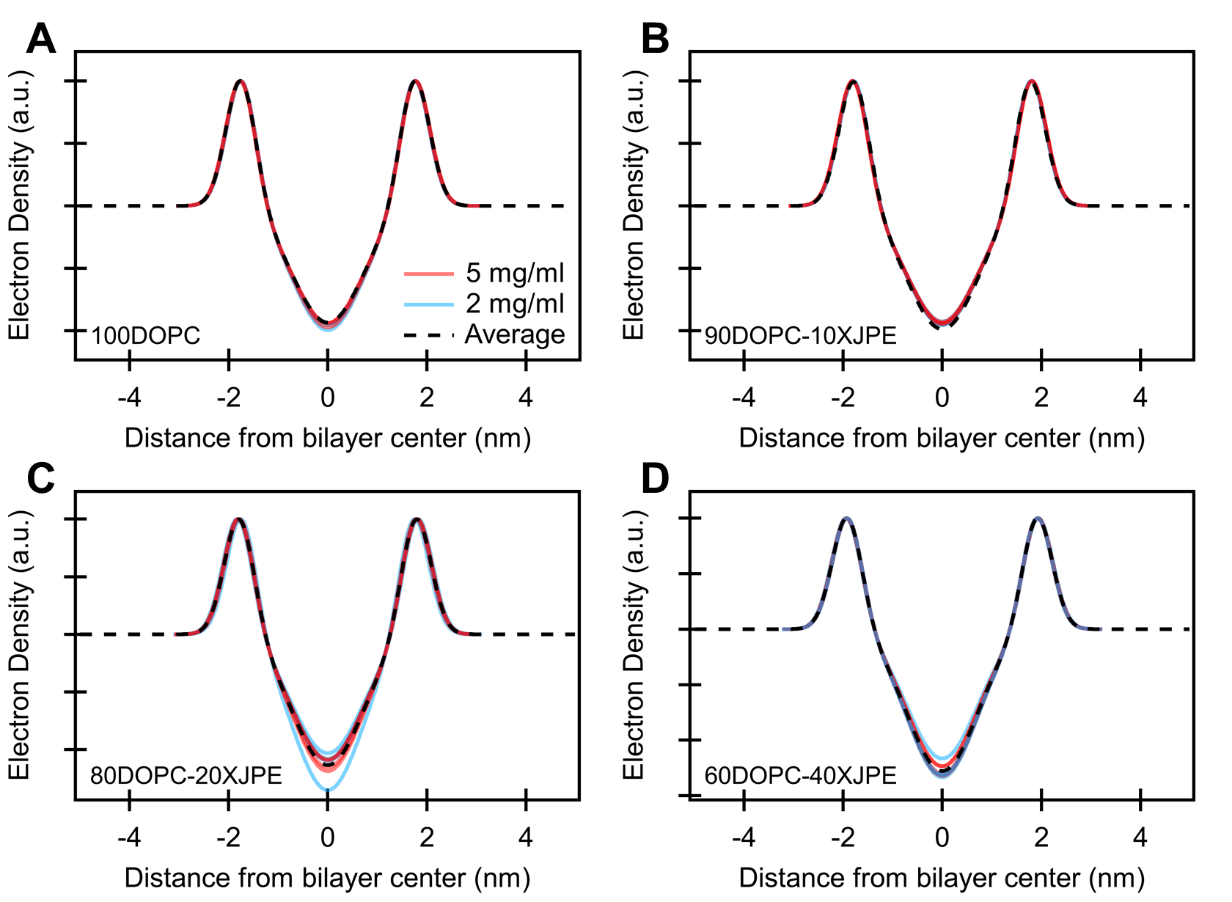


**Figure S15.** Symmetric electron density profiles from scattering models displayed in Figure S11 for DOPC with **(A)** 0%, **(B)** 10%, **(C)** 20%, and **(D)** 40% XJPE. All profiles are overlayed to demonstrate differences between individual models. The marker legends in Panel **A** are used in all panels.


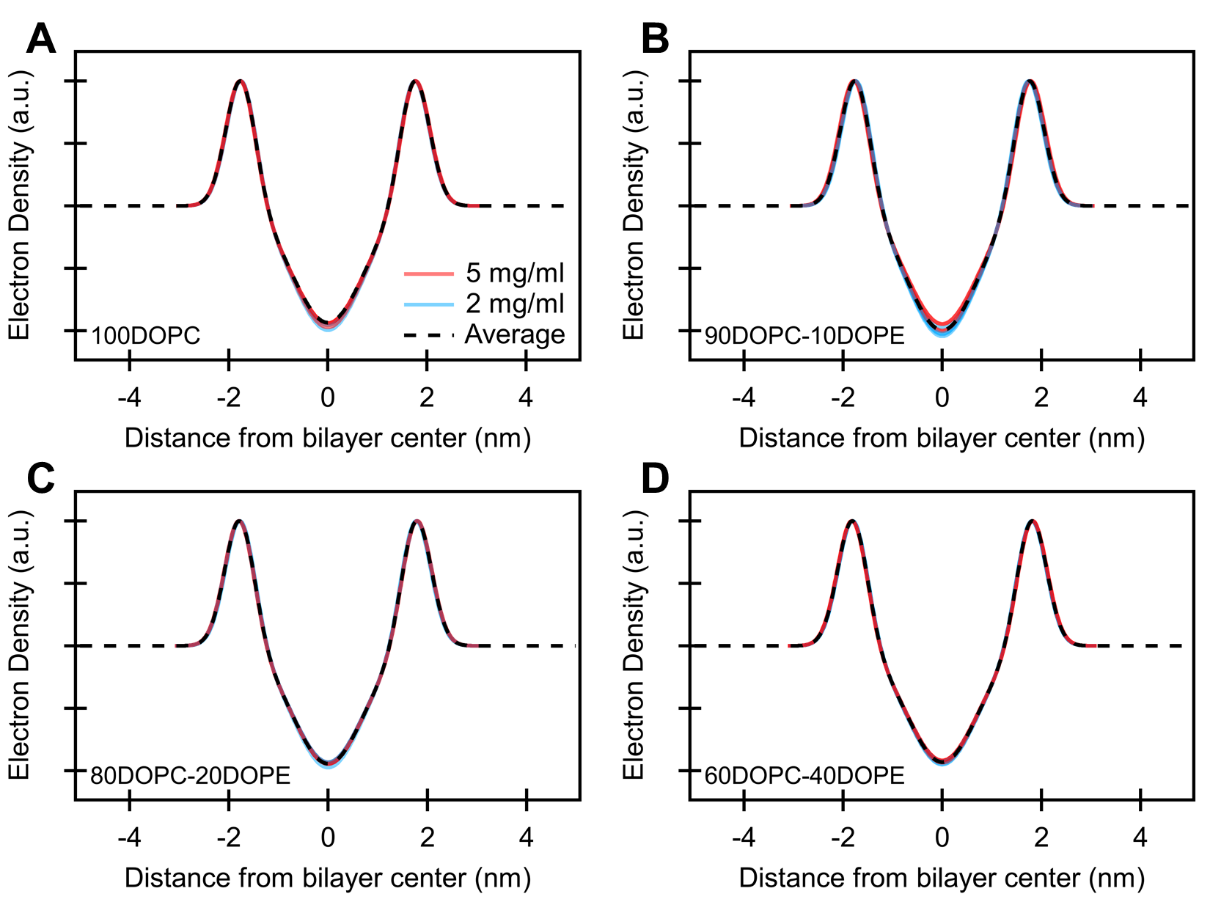


**Figure S16.** Asymmetric electron density profiles from scattering models displayed in Figure S12 for DOPC with **(A)** 0%, **(B)** 10%, **(C)** 20%, and **(D)** 40% DOPE. All profiles are overlayed to demonstrate differences between individual models. The marker legends in Panel **A** are used in all panels.


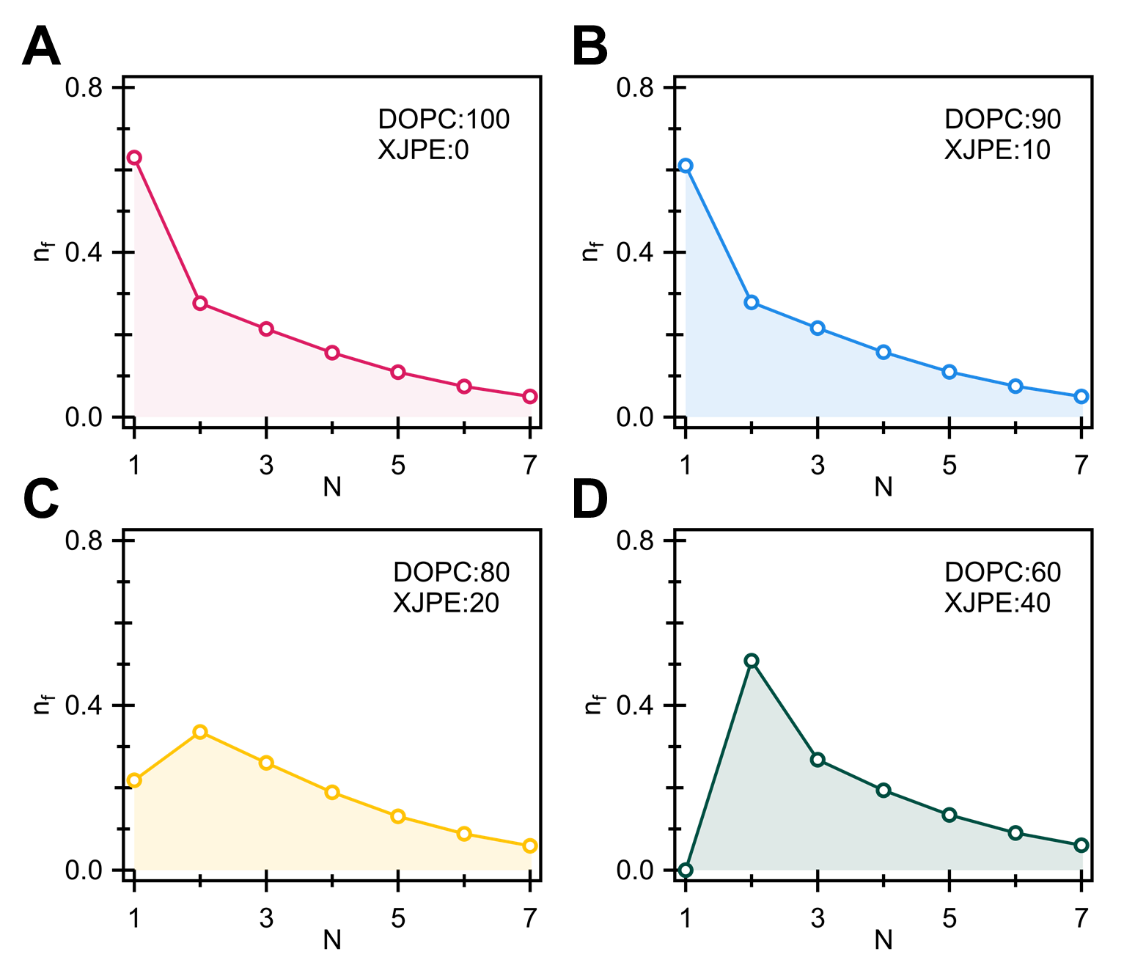


**Figure S17.** Number fraction of multilamellar liposomes (n_f_) is plotted against the number of bilayers (N). Results are extracted from the structure factor of DOPC liposomes with **(A)** 0%, **(B)** 10%, **(C)** 20%, and **(D)** 40% XJPE.


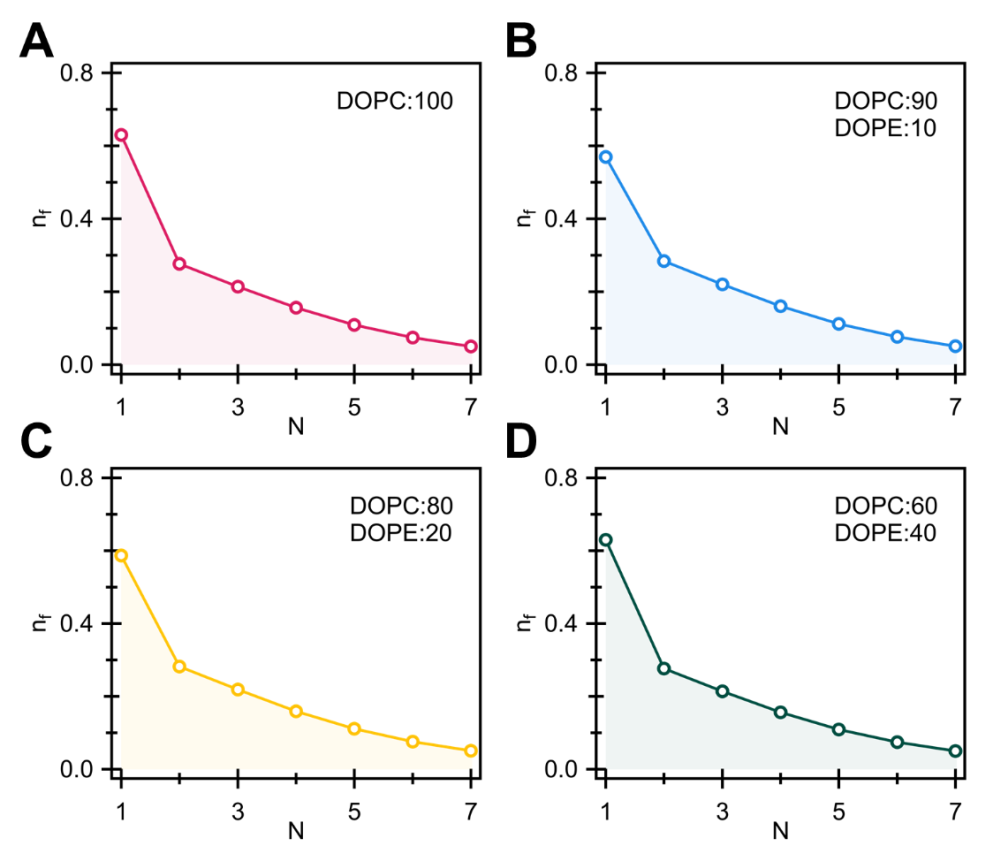


**Figure S18.** Number fraction of multilamellar liposomes (n_f_) is plotted against the number of bilayers (N). Results are extracted from the structure factor of DOPC liposomes with **(A)** 0%, **(B)** 10%, **(C)** 20%, and **(D)** 40% DOPE.


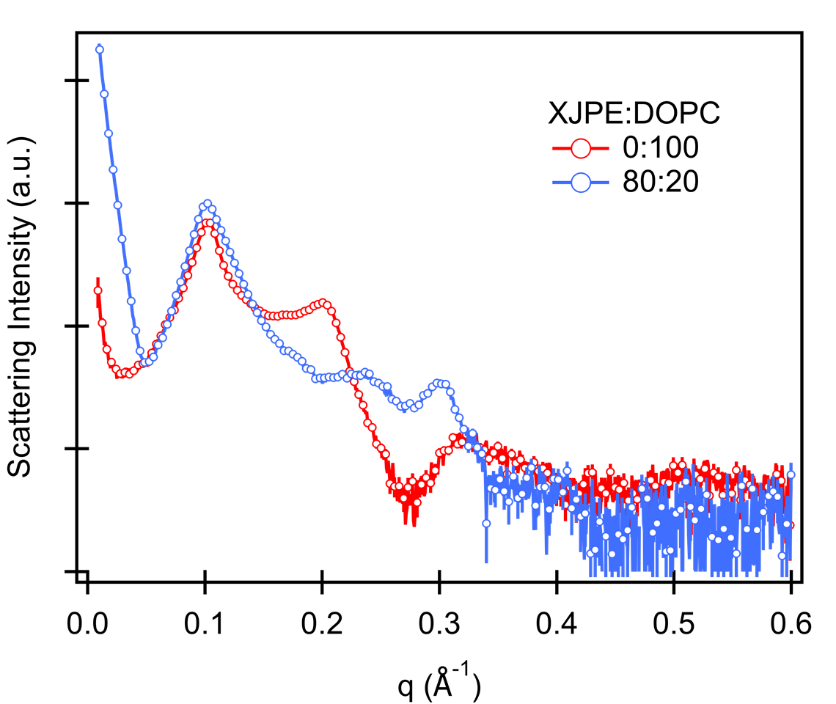


**Figure S19.** Comparison of SAXS data from samples composed of DOPC and XJPE with the ratios given in the figure legend. The 100% DOPC sample exhibits the anticipated form-factor of an MLV while the sample with XJPE exhibits a more complex phase behavior.

**References**

1. Van Der Spoel, D., et al., *GROMACS: Fast, flexible, and free.* Journal of Computational Chemistry, 2005. **26**(16): p. 1701-1718.

2. de Jong, D.H., et al., *Martini straight: Boosting performance using a shorter cutoff and GPUs.* Computer Physics Communications, 2016. **199**: p. 1-7.

3. Smith, P. and C.D. Lorenz, *LiPyphilic: A Python Toolkit for the Analysis of Lipid Membrane Simulations.* Journal of Chemical Theory and Computation, 2021. **17**(9): p. 5907-5919.

4. Bernhardt, N. and J.D. Faraldo-Gómez, *MOSAICS: A software suite for analysis of membrane structure and dynamics in simulated trajectories.* Biophysical Journal, 2023. **122**(11): p. 2023-2040.

5. Torres-Sánchez, A., J.M. Vanegas, and M. Arroyo, *Examining the Mechanical Equilibrium of Microscopic Stresses in Molecular Simulations.* Physical Review Letters, 2015. **114**(25): p. 258102.

6. Vanegas, J.M., A. Torres-Sánchez, and M. Arroyo, *Importance of Force Decomposition for Local Stress Calculations in Biomembrane Molecular Simulations.* Journal of Chemical Theory and Computation, 2014. **10**(2): p. 691-702.

7. Humphrey, W., A. Dalke, and K. Schulten, *VMD: Visual molecular dynamics.* Journal of Molecular Graphics, 1996. **14**(1): p. 33-38.

8. *cg_bonds_v5.tcl* [cited 2024 11/11/24]; Available from: <https://cgmartini.nl/docs/downloads/tools/visualization.html>.

9. Marquardt, D., B. Geier, and G. Pabst, *Asymmetric Lipid Membranes: Towards More Realistic Model Systems.* Membranes, 2015. **5**(2): p. 180-196.

10. Frewein, M.P.K., et al., *Structure and Interdigitation of Chain-Asymmetric Phosphatidylcholines and Milk Sphingomyelin in the Fluid Phase.* Symmetry, 2021. **13**(8): p. 1441.

11. Pencer, J., et al., *Method of separated form factors for polydisperse vesicles.* Journal of applied crystallography, 2006. **39**(3): p. 293-303.

12. Pencer, J. and F.R. Hallett, *Small-angle neutron scattering from large unilamellar vesicles: An improved method for membrane thickness determination.* Physical Review E, 2000. **61**(3): p. 3003-3008.

13. Zhang, R., R.M. Suter, and J.F. Nagle, *Theory of the structure factor of lipid bilayers.* Physical Review E, 1994. **50**(6): p. 5047-5060.

14. Konarev, P.V., et al., *Restoring structural parameters of lipid mixtures from small-angle X-ray scattering data.* Journal of applied crystallography, 2021. **54**(1): p. 169-179.

15. Scott, H.L., et al., *On the Mechanism of Bilayer Separation by Extrusion, or Why Your LUVs Are Not Really Unilamellar.* Biophysical Journal, 2019. **117**(8): p. 1381-1386.
